# Carotenoids for Antiaging: Nutraceutical, Pharmaceutical, and Cosmeceutical Applications

**DOI:** 10.3390/ph18030403

**Published:** 2025-03-13

**Authors:** Mariia Shanaida, Olha Mykhailenko, Roman Lysiuk, Nataliia Hudz, Radosław Balwierz, Arkadii Shulhai, Nataliya Shapovalova, Volodymyr Shanaida, Geir Bjørklund

**Affiliations:** 1 Department of Pharmacognosy and Medical Botany, I. Horbachevsky Ternopil National Medical University, 46001 Ternopil, Ukraine; 2 CONEM Ukraine Natural Drugs Research Group, 46001 Ternopil, Ukraine; shanayda-vv@ukr.net; 3 Department of Pharmaceutical Chemistry, National University of Pharmacy, 61168 Kharkiv, Ukraine; o.mykhailenko@nuph.edu.ua; 4 Department of Pharmacognosy and Botany, Danylo Halytsky Lviv National Medical University, 79010 Lviv, Ukraine; pharmacognosy.org.ua@ukr.net (R.L.); tatamed@ukr.net (N.S.); 5 CONEM Ukraine Life Science Research Group, 79010 Lviv, Ukraine; 6 Department of Drug Technology and Biopharmacy, Danylo Halytsky Lviv National Medical University, 79010 Lviv, Ukraine; natali_gudz@ukr.net; 7 Department of Pharmacy and Ecological Chemistry, University of Opole, 45-052 Opole, Poland; radoslaw.balwierz@gmail.com; 8 Department of Public Health and Healthcare Management, I. Horbachevsky Ternopil National Medical University, 46001 Ternopil, Ukraine; shulhaiag@tdmu.edu.ua; 9 Design of Machine Tools, Instruments and Machines Department, Ternopil Ivan Puluj National Technical University, 46001 Ternopil, Ukraine; 10 Council for Nutritional and Environmental Medicine, 8610 Mo i Rana, Norway

**Keywords:** age-related disorders, *α*-carotene, *β*-carotene, lycopene, lutein, astaxanthin, *β*-cryptoxanthin, fucoxanthin, zeaxanthin, pharmacological effects, antiaging properties, possible toxic effects

## Abstract

**Background**: Carotenoids are bioactive tetraterpenoid C40 pigments that are actively synthesized by plants, bacteria, and fungi. Compounds such as *α*-carotene, *β*-carotene, lycopene, lutein, astaxanthin, *β*-cryptoxanthin, fucoxanthin, and zeaxanthin have attracted increasing attention for their antiaging properties. They exhibit antioxidant, neuroprotective, and anti-inflammatory properties, contributing to the prevention and treatment of age-related diseases. **Objectives**: The aim of this study was to comprehensively analyze the pharmacological potential and biological mechanisms of carotenoids associated with age-related disorders and to evaluate their application in nutraceuticals, pharmaceuticals, and cosmeceuticals. **Methods**: A systematic review of studies published over the past two decades was conducted using the databases PubMed, Scopus, and Web of Science. The selection criteria included clinical, in silico, in vivo, and in vitro studies investigating the pharmacological and therapeutic effects of carotenoids. **Results**: Carotenoids demonstrate a variety of health benefits, including the prevention of age-related macular degeneration, cancer, cognitive decline, metabolic disorders, and skin aging. Their role in nutraceuticals is well supported by their ability to modulate oxidative stress and inflammatory pathways. In pharmaceuticals, carotenoids show promising results in formulations targeting neurodegenerative diseases and metabolic disorders. In cosmeceuticals, they improve skin health by protecting it against UV radiation and oxidative damage. However, bioavailability, optimal dosages, toxicity, and interactions with other bioactive compounds remain critical factors to maximize therapeutic efficacy and still require careful evaluation by scientists. **Conclusions**: Carotenoids are promising bioactive compounds for antiaging interventions with potential applications in a variety of fields. Further research is needed to optimize their formulas, improve bioavailability, and confirm their long-term safety and effectiveness, especially in the aging population.

## 1. Introduction

People today live longer than ever before; however, these extended lifespans are often accompanied by an increased burden of late-life diseases [1]. Aging occurs due to the accumulation of various forms of molecular and cellular damage in the body over time. This accumulation of damage increases the risk of age-related diseases, including cardiovascular conditions, neurodegenerative disorders, cancer, metabolic syndromes, and other chronic illnesses [2]. Oxidative stress, a significant contributor to aging, worsens this process through diverse mechanisms [3].

Aging is characterized by chronic inflammation, immune system dysfunction, and an imbalance between pro-oxidants and antioxidants, collectively leading to heightened oxidative stress [4]. These processes are strongly associated with cognitive decline, psychological impairment, and physical frailty [5]. Generally, aging is a unique and inevitable biological process characterized by the degeneration of biological components and the disruption of physiological functions in cells and tissues [6].

Elevated levels of reactive oxygen species (ROS) and reactive nitrogen species (RNS), produced from both internal sources (such as mitochondria and NADPH oxidases) and external sources (including certain drugs, cigarette smoke, food, radiation, and pollution), lead to a harmful condition known as oxidative stress [7]. While it is widely recognized that many chronic diseases have multiple contributing factors, they all share oxidative stress as a common underlying factor. Oxidative stress is considered a key factor in the development and progression of various chronic diseases, and it plays a significant role in human skin aging and dermal damage [8]. According to their origin, antioxidants could be internal (endogenous) and external (exogenous) [9]. The latter enter the body from the outside and can be of both natural and synthetic origin. Antioxidants play a vital role in reducing oxidative stress, which can lead to cellular damage. Antioxidants can interrupt radical chain reactions and quench ROS/RNS due to two primary mechanisms: single electron transfer and hydrogen atom transfer reactions [7,8]. This ability allows antioxidants to neutralize free radicals, effectively addressing their unpaired state. As a result, they play an important role in combating age-related and degenerative diseases associated with oxidative stress.

The World Health Organization (WHO) has identified the aging of the global population as one of the most significant demographic challenges worldwide. Consequently, promoting healthy aging has become increasingly critical to maintaining functional abilities [10]. Addressing cognitive decline, psychological impairment, and physical frailty is a major focus of gerontology. Current aging research focuses on developing strategies to mitigate the detrimental effects of aging. In this regard, the term ‘geroprotector’ has gained importance, referring to molecules that target the biological hallmarks of aging, delay the onset of age-related diseases, and enhance resilience in older adults.

According to WHO data, global life expectancy increased from 66.8 years in 2000 to 73.1 years in 2019. However, healthy life expectancy increased from 58.1 in 2000 to 63.5 in 2019. This indicates that the increase in healthy life expectancy (5.3 years) has not kept pace with the overall rise in life expectancy (6.4 years) [11]. These trends underscore the urgent need for interventions that improve both the quality and duration of life.

Free radicals, including ROS/RNS such as superoxide and singlet oxygen, as well as lipid peroxyl radicals, are major contributors to the development of non-communicable diseases. External factors such as smoking, environmental pollution, ultraviolet (UV) exposure, mutagens, and drugs significantly increase ROS levels, resulting in oxidative stress that damages cellular structures and functions [12,13,14]. Non-communicable diseases have notably contributed to higher morbidity and mortality rates, especially in middle- and low-income countries [13,15]. This category of diseases includes cardiovascular diseases (17.9 million people annually), cancer (9.3 million people annually), chronic respiratory diseases (4.1 million people annually), and diabetes (2.0 million, including kidney disease deaths caused by diabetes) [15,16]. Chronic obesity is a direct cause or a major risk factor for the development of many diseases, such as hypertension, dyslipidemia, kidney disease, type 2 diabetes, sleep disorders, and various cancer types [17].

For decades, researchers have been studying ways to slow down aging through various methods, including medical treatments, lifestyle changes, and social programs [18]. The field of aging research is now entering a transformative era, with profound implications for both medical science and societal well-being. This evolution marks a critical juncture not only for aging research but for all biological research that impacts human health and longevity [19]. To mitigate the incidence of these diseases and improve human health, finding ways to combat this damage is essential. In this context, geroprotectors have been proposed as compounds capable of slowing or preventing age-related disorders by targeting key mechanisms of aging and promoting cellular resilience [2].

Slowing down the process of aging may involve using compounds for both dietary and pharmacological purposes to reach maximum lifespan or manage age-related diseases [20]. Natural compounds with radical-scavenging and anti-inflammatory effects, primarily plant-based nutraceuticals, are preferred for the prophylaxis or treatment of age-related chronic diseases. Incorporating these compounds into the diet or utilizing them in pharmaceutical formulations is essential for extending lifespan and improving quality of life [21].

Nutraceuticals are a group of bioactive compounds that align with the need to prevent diseases and promote health. Naturally present in food, these biologically active molecules exhibit properties such as antioxidant, anti-inflammatory, immunomodulatory, and antitumor effects [2]. Nutrients that provide energy—such as lipids, carbohydrates, and proteins—are known as macronutrients. Conversely, vitamins and other organic compounds that are needed in trace amounts and do not directly contribute to energy metabolism are referred to as micronutrients or bioactive compounds [22]. In recent decades, micronutrients have been linked to overall well-being and health benefits associated with disease prevention and treatment [2]. Recently, *β*-carotene, lutein, *α*-lipoic acid, caffeine, curcumin, resveratrol, and some vitamins were named among the most studied antiaging compounds [20,21,23,24,25,26]. These compounds have shown promise as both nutraceuticals—targeting health and disease prevention—and cosmeceuticals, addressing skin aging and related cosmetic concerns.

Carotenoids are red, orange, and yellow pigments widespread in nature. With a few rare exceptions, animals cannot synthesize carotenoids and must obtain them by consuming plants [27]. Carotenoids are colorful C40 tetra-terpenoid pigments produced by various plants, bacteria, and fungi [28,29,30]. The most significant carotenoids in the human diet were discovered in various plants by the mid-20th century and include carotene from carrots, lycopene from tomatoes, lutein from leafy greens, cryptoxanthin from papayas, and zeaxanthin from corn. In recent decades, carotenoids were isolated from animal and human biofluids and tissues, including plasma, placenta, milk, corpus luteum, and the macula lutea. Although there are a thousand carotenoids in nature, only a few are essential in the human diet [31]. Generally, only a few carotenoids are commonly found in the human diet and serum. The primary carotenoids present in the human diet and serum include *α*-carotene, *β*-carotene, lutein, lycopene, zeaxanthin, and *β*-cryptoxanthin [32]. These compounds exhibit a range of health-promoting properties, making them promising candidates for treating age-related diseases.

Carotenoids have various biological activities, such as radical scavenging properties, anti-inflammatory effects, and provitamin A activity, which make them attractive for numerous applications in medicine. Lutein, *β*-carotene, and zeaxanthin deserve special attention because of their exclusive accumulation at high concentrations in the macula lutea of the retina. This accumulation helps protect against age-related macular degeneration (AMD) by reducing light-induced oxidative stress [33].

Various stress factors, including illnesses, UV and infrared (IR) radiation from the sun, smoking, and alcohol consumption, can lower the levels of carotenoids in the skin. Patients who have higher levels of carotenoids and other antioxidants in their tissues tend to experience less premature skin aging [34].

In recent years, the colorless 40-carbon phytofluene and phytoene, which intermediate in the biosynthesis of other carotenoids and are available mainly from tomato, have attracted increased interest in nutrition and cosmetics due to their UV radiation-absorbing properties [35]. Thus, the extract from golden tomatoes used in a study of dermal fibroblasts under oxidative stress primarily contained phytoene (6.7%) and phytofluene (1.9%) as the main carotenoids. It also included smaller amounts of *β*-carotene (0.14%) and lycopene (0.1%) [36].

The National Chinese Health and Nutrition Examination Survey, involving 19,280 participants, investigated the relationship between dietary carotenoid levels and biological age [37]. It was found that participants with higher carotenoid intake experienced lower rates of phenotypic age acceleration. Specific carotenoids, including *α*-carotene, *β*-carotene, *β*-cryptoxanthin, zeaxanthin, lutein, and lycopene, exhibited protective effects against senescence. The research study suggests the potential of dietary carotenoids in slowing biological aging and underscores their protective role in the aging process. The economic significance of carotenoids has also grown substantially. In 2019, the global market value of carotenoids was estimated at USD 1.44 billion, with projections indicating an increase to over USD 1.84 billion by 2027, reflecting an annual growth rate of 3.4%. The current market is increasingly focused on sourcing carotenoids from natural origins for applications in cosmetics and nutraceuticals [38].

The objective of this study is to examine the impact of carotenoids on the aging process, with a particular focus on their role in protecting against oxidative stress-induced disorders and UV and IR radiation, as well as their potential utilization. The analysis encompasses the relationship between carotenoid consumption and the acceleration of biological age, in addition to their beneficial effects on health, including the prevention of chronic diseases such as macular degeneration, cancer, neurodegeneration, and obesity, and the enhancement of skin condition and cardiovascular function. Generally, this study aimed to re-evaluate carotenoids as promising nutraceuticals, pharmaceuticals, and cosmeceuticals, specifically from an antiaging perspective.

## 2. Materials and Methods

The literature search was performed using the PubMed, Scopus, and Web of Science databases. The selection criteria included peer-reviewed articles, clinical studies, meta-analyses, and relevant book chapters published over the last two decades (2004–2024).

The search strategy involved using keywords such as carotenoids, antiaging, oxidative stress, inflammation, nutraceuticals, pharmaceuticals, cosmeceuticals, *β*-carotene, lutein, lycopene, astaxanthin, zeaxanthin, and fucoxanthin. Among the inclusion criteria were studies that (1) investigated the biological activities and health benefits of carotenoids; (2) provided experimental evidence (in vitro, in vivo, in silico, or clinical trials) on carotenoid-mediated antiaging effects; (3) explored applications in nutraceutical, pharmaceutical, or cosmeceutical formulations; (4) were published in English.

Among the exclusion criteria were (1) non-peer-reviewed articles, editorials, and conference abstracts, as well as (2) studies with insufficient methodological details.

## 3. Results

### 3.1. Diversity and Health-Promoting Effects of Carotenoids

Up to now, more than 1100 carotenoid structures have been identified from hundreds of different sources [39,40]. In plants and cyanobacteria, carotenoids play a major role in absorbing energy for photosynthesis and photoprotection within plant tissues. Their role in photoprotection arises from their ability to quench and deactivate ROS, such as singlet oxygen, which is produced when plants are exposed to air and light. Therefore, carotenoids are associated with antioxidant activity that benefits human health [41]. They can react with free radicals, which can lead to the formation of carotenoid radicals. The reactivity of these compounds is influenced by the length of the chain of conjugated double bonds and the properties of their end groups. Carotenoid radicals are stable due to the delocalization of the unpaired electron across the conjugated polyene chain. This delocalization also enables further reactions to take place at multiple sites on the radical.

**Figure 1:**
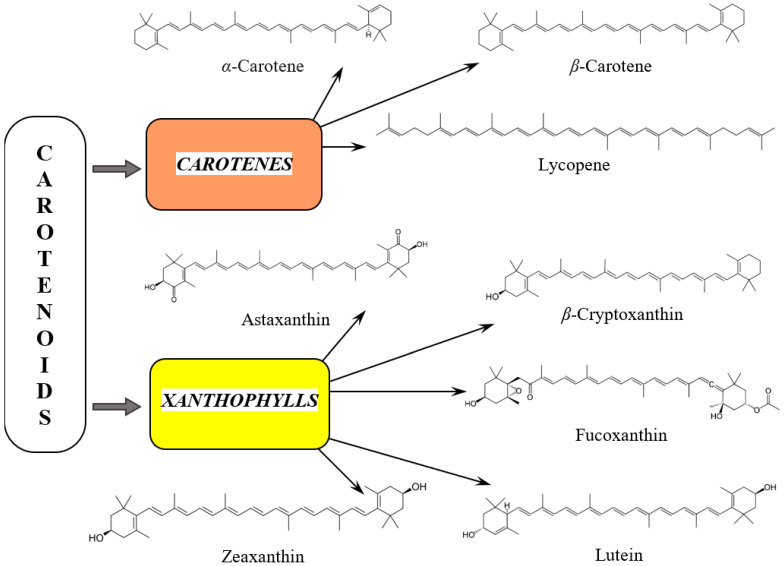
The most common representatives of carotenoids.

Carotenoids come in two classes (Figure 1): carotenes, which are pure hydrocarbons, and xanthophylls, which are oxygenated molecules. In plants, carotenoids play a major role in absorbing energy for photosynthesis and protecting chlorophyll from damage. The main examples of carotenes include *α*- and *β*-carotenes and lycopene. The xanthophyll category mainly comprises lutein, *β*-cryptoxanthin, fucoxanthin, astaxanthin, and zeaxanthin [25].

Carotenoids are synthesized by phototrophs (such as cyanobacteria, algae, and higher plants), non-photosynthetic bacteria, and fungi [25]. The structure of carotenoids can be described as tetraterpenes with eight five-carbon isoprenoid units (mainly C40). They can contain up to 15 conjugated double bonds and exhibit a symmetric arrangement, typified by *β*-carotene, resulting in red, orange, or yellow colors. All carotenoids exist in two isomeric configurations, *trans* and *cis*, due to the presence of conjugated double bonds in their polyene chains. Carotenoid compounds are predominantly found in nature in their all-*trans* form [41]. Interestingly, the *cis* form of fucoxanthin has lower antioxidant activity compared to its all-trans form [42,43,44].

Different carotenoids have been revealed in diverse natural sources, including brown algae and microalgae such as *Dunaliella salina*, *Heterochlorella luteoviridis*, or *Chlorella vulgaris*, by-products of carrot juice, tomatoes, maize grains, apricot waste, citrus residue, pumpkin pulp, sea buckthorn berries, red pepper, *Calendula officinalis* flowers, and yeast such as *Rhodotorula glutinis* or *Xanthophyllomyces*, among others. These abundant sources offer promising prospects for the extraction of carotenoids, which are applicable not only in traditional industries such as food dyes, nutraceuticals, and cosmetics but also in the pharmaceutical industry [39].

The carotenoid content in natural sources can vary substantially due to genetic, environmental, and harvesting factors [45]. The changes in carotenoid biosynthesis caused by external environmental factors, such as high levels of drought and chilling stresses, led to significantly impact the nutritional value of crop plants. Additionally, various cultivation practices also play a crucial role in determining the content of carotenoids in crops. Recent research conducted by Popa and colleagues [46] found that the total carotenoid levels including zeaxanthin, *β*-cryptoxanthin, lutein, and *β*-carotene in the grains of several modern *Zea mais* hybrids ranged from 7.45 to 25.08 μg/g.

Carotenoids play a crucial role in maintaining various functions of the human body. These include promoting eye, skin, and cardiovascular health and supporting immunity, cognitive function, and bone health. They work by neutralizing free radicals, regulating inflammatory pathways, and modulating gene expression [39,47]. Carotenoids (*α*-carotene, *β*-carotene, and *β*-cryptoxanthin) are precursors to vitamin A and retinoids as a result. They can be metabolized into shorter apo-carotenoids such as retinol. Lutein and zeaxanthin, in particular, have been found to help prevent AMD, which is the primary cause of vision loss in the elderly [48].

**Figure 2:**
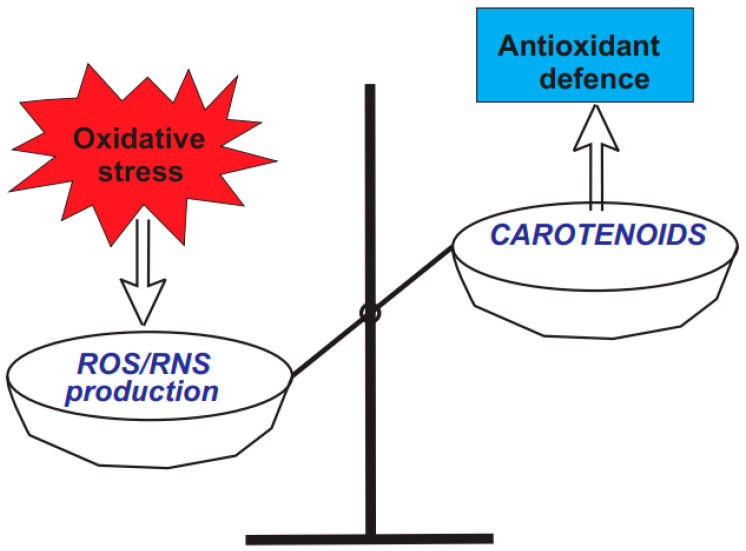
Leading mechanism contributing to the antiaging effects of carotenoids.

Carotenoids play crucial roles in human overall health, primarily due to their well-known antioxidant effects (Figure 2) [49]. The ability of seven natural carotenoids to scavenge hydroxyl radicals (HO•), peroxyl radicals (ROO•), hypochlorous acid (HOCl), and peroxynitrite (ONOO−) was assessed in liposomes [50]. The application of liposomes as model membrane systems to assess the antioxidant capacity of bioactive carotenoids has gained interest due to the structural similarities between liposomes’ bilayer and the lipid composition of cell membranes. The initial two reactive species (HO• and ROO•) are particularly important due to their significant role in lipid peroxidation, which is associated with the onset of atherosclerosis and other cardiovascular diseases [50].

Three mechanisms have been proposed for the scavenging free radicals by carotenoids: electron transfer, abstraction of the allylic hydrogen, and radical addition to the system of conjugated double bonds [51]. The presence of conjugated double bonds allows carotenoids to accommodate unpaired electrons, thereby neutralizing free radicals [52]. The specific mechanism that occurs depends on the organization level of the reaction system, its polarity, and the structure of the particular carotenoid. Interestingly, among the carotenoids tested, astaxanthin proved to be the most effective scavenger of ROS, whereas *β*-carotene was identified as the most potent scavenger of RNS [50].

A large body of data links carotenoid intake to the prevention of aging-related conditions and diseases [53]. They are linked to a reduced risk of various chronic diseases. Research suggests that their dietary intake and circulating levels are associated with a lower incidence of type 2 diabetes, obesity, certain types of cancer, and overall lower mortality rates. Carotenoids may protect skeletal muscle from sarcopenia, which is the age-related loss of muscle mass and function. They can modulate genes expression and regulate cell growth as well as and immune responses [54].

As is well known, managing metabolic syndrome involves using numerous medications, which, despite being effective, can be challenging due to their high costs, prolonged use, and various side effects. Polyphenols and carotenoids have been suggested as alternative treatments for metabolic syndrome because they can improve insulin response, lower serum triglyceride concentrations, inhibit adipogenesis, and downregulate angiotensin-converting enzyme activity [55,56]. Some carotenoids are also precursors to vitamin A and are thought to have potential antioxidant effects, as well as being involved in pathways related to inflammation and oxidative stress [48]. *β*-carotene and lycopene are the most abundant carotenoids in nature [57].

The levels of collagen, which is the primary structural protein in the body, decline with age, leading to a loss of skin elasticity as a hallmark of aging skin [58]. Several factors contribute to collagen degradation; examples include hormonal disbalance, environmental factors such as UV radiation, smoking, inappropriate dietary habits, and chronic stress [59]. Carotenoids in the skin can neutralize ROS generated by environmental stressors, potentially reducing oxidative damage to collagen [60]. It is worth noting that collagen production, particularly type I collagen, can be mechanically stimulated [59]. Thus, combining carotenoid supplementation with mechanical stimulation methods appears to be a rational approach to maintaining collagen integrity and promoting skin health [61].

It was found that the natural carotenoid-rich extract from curly kale (dosage: 1.65 mg of carotenoids in total, once a day) could effectively prevent aging-related collagen degradation in the dermis of healthy female volunteers [60]. These results showed significantly increased values for the collagen I/elastin aging index of the dermis and the cutaneous carotenoids levels after 5 and 10 months of extract administration. As was revealed recently, carotenoids and other natural compounds are able to protect human dermal fibroblasts, which are the main source of collagen in the skin, from ROS-induced damage [62]. Subsequently, it was proven that this combination effectively protected dermal fibroblasts from cell death, senescence, and deterioration of collagen homeostasis induced by mitochondrial dysfunction [36]. Carotenoid-rich extracts positively impacted collagen biosynthesis and are considered evidence-based dietary compounds for skin aging therapy [36].

As is known, several mechanisms contribute to the protection of skin health; these include antioxidant, anti-inflammatory, and photoprotective effects, as well as collagen formation and reduction in matrix metalloproteinases [63,64]. A decrease in collagen density, which is a key structural protein, leads to reduced elasticity and gradual skin atrophy. Increases in matrix metalloproteinases and lysyl oxidase concentrations over time result in the accumulation of fragmented and cross-linked collagen [63]. The processes of photoaging contribute to collagen fragmentation and hinder its formation. The topical application of carotenoid-related substances, found in over-the-counter or cosmeceutical products, has shown promise in increasing collagen and elastic fibers, inhibiting collagen-degrading enzymes, and normalizing the function of melanocytes and keratinocytes [65].

It was revealed that pancreatic insufficiency is common in individuals with cystic fibrosis and can lead to a decreased absorption of carotenoids, even when they receive pancreatic enzyme replacement therapy [66]. Since the macular pigment in the retina is primarily composed of lutein and zeaxanthin, lower serum levels of these nutrients in people with cystic fibrosis may adversely affect their ocular and retinal health.

Due to the hydrophobic properties of carotenoids, they are absorbed in the intestines of the human/mammalian digestive tract along with dietary fats [49]. Moreover, the bioavailability and delivery of carotenoids can be significantly improved when they are suspended in a mixture of extra virgin olive oil and other vegetable oils [67].

Carotenoids, which are fat-soluble micronutrients, behave similarly to lipids in the human upper digestive tract [68]. The first step in their digestion involves their dissolution in fat from the meal. This fat phase is then emulsified into lipid droplets in the stomach and duodenum. During the digestion process in the duodenum, carotenoids combine with other lipids, such as cholesterol or phospholipids, as well as lipid digestion products like free fatty acids, monoacylglycerols, and lysophospholipids, to form mixed micelles. Additionally, some carotenoids may associate with proteins during this process.

Carotenoids can be absorbed by the intestine through passive diffusion or a transporter-dependent process in the enterocytes’ membrane. Variations in certain genes within the human population can influence how individuals respond to carotenoid intake [69]. In the future, it will be important to consider whether someone is a ‘low responder’ or ‘high responder’ to carotenoids, which can result from differences in transport and conversion efficiency. This approach could allow for personalized dietary recommendations aimed at optimizing the health benefits of carotenoids for each individual.

**Figure 3:**
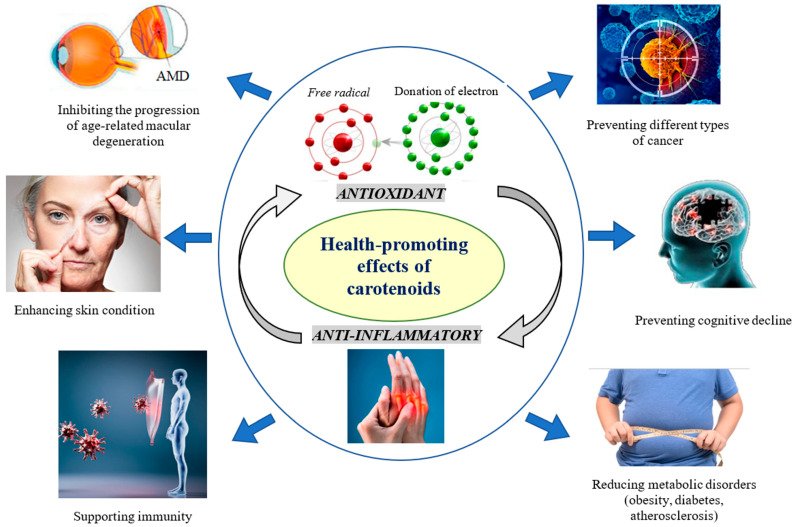
Therapeutic potential of carotenoids in relation to antiaging benefits.

Carotenoids can inhibit the development of AMD, prevent cancer, reduce obesity, prevent neurodegeneration, enhance skin functions, etc. (Figure 3) [13,17,32,70]. Recently, a relationship was found between the levels of serum carotenoids and telomere length in obese individuals [71].

To ensure the optimal delivery and bioavailability of pigments such as lutein and zeaxanthin, these compounds should be encapsulated in a compatible vegetable oil-based liposome, due to the presence of long-chain aliphatic molecules. Given that carotenoids have low bioavailability due to their lipophilicity and low stability as a result of their conjugated double bonds, nanoscale delivery systems have recently been proposed for their controlled delivery to a target [72]. Marine carotenoids often exhibit low stability, poor absorption, and limited bioavailability [73]. A promising solution to these challenges is nanoencapsulation, a technique that helps preserve marine carotenoids and their original properties during processing and storage. Recent data on various carotenoids (lycopene, lutein, astaxanthin, fucoxanthin, crocin, etc.) that are used for the treatment of Alzheimer’s disease showcase the potential of nano-drug delivery, including lipid, polymer, inorganic, and hybrid nanoformulations [74]. A microfluidic technique was developed to create a system for nanoencapsulating astaxanthin, which significantly improves its bioaccessibility [75].

The in silico screening of 27 carotenoids isolated from green algae identified them as natural inhibitors of proprotein convertase subtilisin/kexin type 9 (PCSK9) as a key regulator of cholesterol metabolism. These findings provide evidence that specific carotenoids, particularly astaxanthin and siphonxanthin, may provide a potential approach for reducing cardiovascular risk [76]. Carotenoids may protect skeletal muscle from sarcopenia, which is the age-related loss of muscle mass and function. In studies involving mouse myoblasts, lycopene was found to stimulate cell proliferation and enhance mitochondrial ATP production [77].

Generally, there are many chronic diseases, including comorbid conditions, that can be managed with the help of carotenoids [78]. It has been found that diabetic patients are about three times more susceptible to periodontitis due to oxidative stress and increased inflammatory levels caused by high blood sugar levels, leading to damage to the periodontal tissue. Carotenoids are considered effective nutrients for mitigating periodontitis [79]. In a recent study initiated by Li and co-authors [80] in China, a total of 1914 diabetic patients were involved, with 1281 in the periodontitis group. The study revealed that the association between carotenoid intake and periodontitis varied across different subpopulations. After adjusting for age, gender, education, smoking, the number of missing teeth, dental implants, and hepatitis, it was found that lutein and zeaxanthin intake of ≥795.95 μg was associated with decreased odds of periodontitis [80].

### 3.2. Carotenes

#### 3.2.1. *α*-Carotene

*α*-carotene, a dietary provitamin A carotenoid, is a yellow-orange pigment. Chemically, it is a non-oxygenated terpene, based on eight isoprenoid units with the general formula C_40_H_56_ (molecular weight 536.88 g/moL) (Figure 1). Its basic linear and symmetrical skeleton consists of a central position with 22 C atoms and two cyclized terminal units of 9 C atoms, containing a parent hydrocarbon chain without any functional groups [54,81,82,83,84]. It is highly soluble in organic solvents and insoluble in polar ones [85].

*α*-carotene is one of the most common carotenoids included in human nutrition [85] and is typically found in deep green, yellow, orange, or red fruits and vegetables [5,81,86,87]. As *α*-carotene is not produced in the human body, it must be obtained through diet [54,88]. The dietary sources of *α*-carotene include apricots, asparagus, avocados, bell peppers, blackberry, broccoli, carrots, cereal grains, green beans, mango, melon, oranges, papaya, pumpkin, spinach, and tangerines [5,28,82,83,85,86,89,90,91,92,93].

The basic biological function of *α*-carotene in humans is its provitamin A action. It is converted by carotene oxygenase into biologically active vitamin A [54,92,94]. The enzymatic cleavage of *α*-carotene results in the formation of only one retinol molecule, because it consists of one *β*-ionone ring [38,85]. Generally, *α*-carotene, as a provitamin A carotenoid, contains an unsubstituted *β*-ionone ring, converted into retinal, which is essential for the development, maintenance and protection of vision [5,85].

*α*-carotene is one of the approximately 20 carotenoids that were detected in human tissues and serum in the last decades [54,87,88,90,95]. It is mainly stored in adipose and liver tissues [38]. The half-life of *α*-carotene in human plasma is estimated to be 45 days in healthy adult females and less than 12 days in healthy males [92]. Its concentration in serum is 0.075 µmol/L [38] and is correlated with the total intake of fruits, vegetables, or plant oils [85,89,91]. Its bioavailability and ability to be converted into retinol should also be considered [85]. Proper food preparation, including high-temperature cooking, improves the absorption of *α*-carotene by the intestinal mucosa and the formation of complexes with proteins or very-low-density lipoproteins which further circulate throughout the human body [90].

*α*-carotene is known for its antioxidant and immune-enhancing effects and its ability to lower the risk of some types of cancer [86]. *α*-carotene scavenges singlet oxygen, hydrogen peroxide, nitric oxide radical, and peroxynitrite anion due to their π-π-conjugated C=C double bonds, located in the ionone rings. Generally, *α*-carotene promotes free radical quenching, reduces injury from ROS, and inhibits lipid peroxidation, which contributes to protection against chronic ailments [54,85,96]. The low plasma concentration of *α*-carotene is strongly correlated with frailty and higher oxidative stress levels in humans compared with their robust counterparts [87].

The antiaging effect of *α*-carotene has been proven in several clinical studies. For example, in a cohort of 512 late post-menopausal females over 65 years old, it was found that *α*-carotene concentration was significantly and inversely correlated with estradiol level. A statistically significant and positive correlation was also found between testosterone and the testosterone-to-estradiol ratio [97]. A cross-sectional trial involving 1172 individuals aged over 50 years revealed a positive association of *α*-carotene level with muscle strength in older adults and suggested that a higher intake of *α*-carotene containing fruits and vegetables might be a protective measure for muscle strength in elderly individuals [89].

A randomized controlled trial, ‘The Mediterranean-DASH Intervention for Neurodegenerative Delay’, demonstrated that higher plasma *α*-carotene levels (349.0 μg/L), caused by an adequate consumption of fruits and vegetables and a lower intake of butter, margarine, and meat, in a US population with suboptimal nutrition between the ages of 65 and 84 years and at risk for cognitive decline, was associated with improved scores in both semantic memory scores and global cognition. The global cognitive score increased as the plasma *α*-carotene level was elevated from 34.9 to 155.8 μg/L. Participants in the highest tertile of plasma *α*-carotene intake had a global cognition z-score that was 0.17 higher than that of those in the lowest tertile. The study outcomes show the importance of the Mediterranean diet, rich in *α*-carotene, as a promising strategy for the prevention of cognitive decline [90,91].

It was found that an *α*-carotene intake of more than 1.38 mg/day led to an improvement in cognitive function following the research outcomes of a cross-sectional trial involving around 2000 participants [90]. Lower concentrations of carotenoids, including *α*-carotene, in the blood have been detected in patients with dementia in comparison to control subjects. Some researchers suggest that plasma *α*-carotene levels might be a biomarker of Alzheimer’s disease, as they are significantly lower in such patients [90].

The dietary consumption of *α*-carotene was inversely correlated with the risk of type 2 diabetes. Nonlinear associations were established for circulating levels of *α*-carotene, but not for other carotenoids or dietary intake [98]. A subgroup analysis of 14 observational studies indicated a positive role of *α*-carotene in weight loss [95].

Several carotenoids, including *α*-carotene, have demonstrated anti-carcinogenic activity in various tissues [86]. It was found that *α*-carotene at a dose of 2.5 μM caused a significant inhibition of cell invasion, migration, and adhesion of the human hepatocarcinoma cell line in vitro in a dose-dependent manner; its anti-apoptotic activity exceeded that of *β*-carotene at the same concentration [54]. A case–control trial among postmenopausal women demonstrated that breast cancer is 25–35% less common in females with the highest quintile of *α*-carotene, in comparison with those in the lowest quintile. A correlation between a diet rich in carotenoids, including *α*-carotene, and a lower risk for breast and endometrial cancers has been observed [97]. The consumption of *α*-carotene was substantially related to improved breast cancer survival, as demonstrated in a meta-analysis involving 19,450 breast cancer patients. The intake of other non-pro-vitamin A carotenoids did not reveal such benefits [99]. A meta-analysis [99] demonstrated that the intake of *α*-carotene had no impact on breast cancer prognosis when evaluating dietary habits before and after diagnosis. A higher serum level of *α*-carotene was associated with a lower risk of cervical cancer in Chinese women [54]. A higher intake and blood concentration of *α*-carotene was linked to a reduced risk of prostate cancer, excluding its advanced forms [54]. Combined treatment with *α*-carotene, lycopene, and *β*-cryptoxanthin was associated with at least a 26% reduction in the frequency of oral and pharyngeal cancer [54].

Regarding *α*-carotene’s influence on skin health, it acts as a provitamin A carotenoid and an antioxidant [100]. Upon oxidation, it produces vitamin A1 (retinol) [96]. In a double-blind, placebo-controlled, randomized clinical trial involving 24 healthy participants, a dietary supplement with a total carotenoid content of 4.45 mg, including 50 µg *α*-carotene, was administered for 8 weeks. This supplementation resulted in a significant increase in skin carotenoid levels in the 4th and 8th week of administration [101].

The National Health and Nutrition Examination Survey (NHANES; 2015–2016) revealed that among the US population, the daily intake of *α*-carotene is 0.34 mg/day [38]. Since the vitamin A equivalent of *α*-carotene is 900 µg, it might be supplied by 21.6 mg/day of *α*-carotene consumption. This compound and other provitamin A carotenoids serve as the sources of vitamin A for vegans [38].

#### 3.2.2. *β*-Carotene

*β*-carotene is a red-orange lipid-soluble pigment based on eight isoprenoid units with the general formula C_40_H_56 (_molecular weight 536.88 g/moL). Its parent hydrocarbon chain has no functional substitutes (Figure 1) [54,83,84,86,102]. *β*-carotene is highly soluble in organic solvents and insoluble in polar ones [85]. *β*-carotene is the most widespread carotene and the most significant provitamin A carotenoid; its main dietary sources comprise orange and yellow vegetables and fruits and dark green leafy vegetables [5,28,81,82,83,92,103].

*β*-carotene is found in acerola, apricot, asparagus, avocado, banana, broccoli, cabbage, carrot, cherry, cereal grains, common purslane, grapefruit, kale, lettuce, mango, melon, orange, papaya, pineapple, pumpkin, red pepper, spinach, sweet potatoes, strawberry, tangerine, and tomato [5,28,38,54,81,82,83,86,90,104,105]. *β*-carotene is one of the main components of cyanobacterium spirulina, a known immunomodulatory and antioxidant agent with nutritional importance [106]. On an industrial scale, *β*-carotene of natural origin is mostly obtained from microalgae and higher plants. The unicellular green alga *Dunaliella salina* is the richest source of *β*-carotene among microalgae. It can accumulate up to 15% of *β*-carotene, expressed in terms of dry cell weight, and is widely applied in its commercial production [38,107]. The filamentous fungus *Blakeslea trispora* is an industrial source of *β*-carotene [38].

The *β*-carotene colorant currently permitted for application in the food industry might be obtained through chemical synthesis or from natural sources (African oil palm and buriti) or by fermentation and bioprocess engineering techniques using algae (*Dunaliella salina*) and fungi (*Blakeslea trispora*) [28]. Recent approaches to *β*-carotene production comprise the application of biofortified crops and microbes, including maize hybrids [38].

It was concluded that steam and microwave cooking had little impact on the carotenoid content of dietary sources, whereas enormous heat can lead to oxidative injury of *β*-carotene [81]. Oven-drying at 50–60 °C preserved 90% of *β*-carotene in sweet potato in comparison with the fresh product, whereas all other techniques (cooking, frying, and sun-drying) reduced the carotenoid content to 30% [85].

The low bioavailability of *β*-carotene in natural sources is caused by the resistance of carotene–protein complexes and plant cell walls to digestion and degradation [5,54,85]. About 5% of carotenoids (whole, raw vegetables) are absorbed by the intestine, whereas up to 10 times more is absorbed from micellar solutions [5]. Interaction with some medications, as well as aspirin and sulphonamides, lowers the bioavailability of *β*-carotene. An age-associated decline in the absorption of various compounds also occurs [5]. Thermal processing, except for extreme heat, enhances the bioavailability and absorption of *β*-carotene compared with mechanical homogenization [5,54]. The bioavailability of *β*-carotene is improved when spinach is ingested in liquefied form in comparison with whole-leaf consumption [104]. Amongst modern technical approaches, the microencapsulation of *β*-carotene enhances its retention, prevents degradation during storage, and, therefore, preserves its antioxidant capacity in comparison with carotenes dissolved in oil. Supercritical carbon dioxide has also been used to encapsulate *β*-carotene [85].

The biological effect of *β*-carotene depends on its source. Solely pure and natural *β*-carotene is easily digestible and has a positive impact on the therapy of ailments, whereas synthetic *β*-carotene does not [107]. Thus, natural *β*-carotene obtained from *Dunaliella salina* developed higher rates of cell mortality in MDA-MB-231 breast cancer cells in comparison with synthetic *β*-carotene [54].

The main pharmacological properties of *β*-carotene include anti-inflammatory, antioxidant, and anticancer effects. It also benefits vision and the cardiovascular system and exhibits immunomodulatory actions [86,107,108]. The concentration of *β*-carotene was lower in children with acute infections compared to healthy subjects [85].

The most significant role of *β*-carotene in humans as a dietary carotenoid is its provitamin A activity, since during absorption in the small intestine, it can release retinol molecules by reacting with *β*-carotene 15,15′-oxygenase within epithelial cells in the presence of oxygen [85,92,94]. This enzyme cleaves *β*-carotene into two *trans*-retinal molecules, which are either oxidized into retinoic acid by enzyme retinal dehydrogenase or reduced into retinol by retinal reductase [38,85,96]. Due to the presence of two unmodified *β*-ionone rings, *β*-carotene within the body has the ability to be converted into vitamin A, an important membrane antioxidant which strengthens the human organism [38,103]. In cases of low vitamin A consumption, the uptake and conversion of *β*-carotene rises. In turn, hypervitaminosis A suppresses the conversion of carotenoids to retinal [90]. Overall, *β*-carotene exhibits a high antioxidant effect and promotes free radical scavenging, effectively removing singlet oxygen, which protects against chronic diseases [85,108].

Several randomized controlled trials in which additional antioxidants such as selenium together with *β*-carotene were administered revealed its beneficial health impact, especially concerning all-cause mortality in men [92,94]. An increased intake of fresh fruits and vegetables is a promising strategy to prevent non-communicable diseases including cancers, diabetes, and cardiovascular and chronic respiratory ailments [92]. The consumption of *β*-carotene prevents low-density lipoprotein (LDL) oxidation, neutralizes peroxide radical formation, and lowers platelet aggregation [85].

In a cortical impact model mimicking traumatic brain damage, the administration of *β*-carotene to mice decreased ROS levels in the brain, assisted by the nuclear accumulation of nuclear factor erythroid 2-related factor 2 (Nrf2) and lowered Keap1 suppression, revealing that *β*-carotene enhanced oxidative stress by modulating the Nrf2/Keap1-mediated pathway [92]. It was found that various combinations of antioxidants, including *β*-carotene and vitamins C and E, act synergistically, significantly expanding the number of neutralized free radicals and thus improving the capability of antioxidant protection [96]. Nevertheless, *β*-carotene can act as a pro-oxidant, contributing to lipid peroxidation’s radical chain reaction in solutions and liposomal membranes at higher oxygen pressures and comparatively high levels [92].

Recently, the antiaging mechanisms of *β*-carotene were studied in vitro using mesenchymal stem cells and in vivo on aged mice. The in vivo research demonstrated that *β*-carotene treatment significantly downregulated the aging state of tissues through the KAT7-p15 signaling axis. Aged mice treated with *β*-carotene revealed improved learning and memory abilities, as well as reduced anxiety [108]. The in vitro study showed that *β*-carotene mitigated mesenchymal stem cell aging, evaluated by aging markers p16 and p21.

A significant lowering in retinol and carotenoid levels occurs in females during aging, as estradiol concentrations decline during menopause and progressively rise in 65+ subjects. In a cohort of late post-menopausal females, the use of a fully adjusted model determined that *β*-carotene was significantly and inversely correlated with estradiol concentrations [97]. Diets rich in carotenoids are related to a lower risk of cardiovascular disorders, AMD, frailty, and other adverse aging outcomes [84,109]. Nutrition low in *β*-carotene might promote frailty risk and is suggested to be a predictive biomarker that turns frailty into disability, since frail and pre-frail participants had significantly lower plasma levels of *β*-carotene, as well as higher degrees of oxidative stress, compared to their robust counterparts in four independent European cohorts [109].

As it was mentioned before, the antiaging potential of carotenoids is mostly caused by their promotion of Nrf2 migration into the nucleus, followed by the transcription of antioxidant and detoxifying enzymes [110]. As a cell divides, its telomere shortens until death, and ROS and inflammation can accelerate telomere diminishing. High dietary *β*-carotene consumption is related to a longer telomere duration.

*β*-carotene exhibits an anti-inflammatory effect on various tissues and cell models, significantly downregulating inflammation-related signaling pathways, as well as on the concentration of inflammatory cytokines such as interleukin (IL) 6 and tumor necrosis factor *alpha* [108]. Supplementation with *β*-carotene suppressed the transcription of IL-1β, IL-6, and IL-12p40 cytokines [54]. *β*-carotene has been suggested as a potential anti-inflammatory drug for DNA virus infections and, especially, for human herpes simplex virus, due to its capability to impede cytokine expression in Suid herpes virus-induced inflammation via nuclear factor kappa B (NF-κB) inactivation. The carotenoid stops the nuclear translocation of the NF-κB p65 subunit, which is associated with its inhibitory impact on the phosphorylation and degradation of the NF-κB inhibitor [54].

The level of *β*-carotene in the serum of Alzheimer’s disease and Parkinson’s disease patients is significantly lower than that of control subjects. In Parkinson’s disease patients, this reduction is related to impaired motor function [81]. Carotenoids may be beneficial in treating Alzheimer’s disease, as long-term placebo-controlled studies indicate that consumption of *β*-carotene can improve cognitive function [90]. A study on the impact of *β*-carotene on cognitive function and verbal memory, which lasted for 18 years and engaged over 4000 participants above 56 years old, demonstrated that its supplementation at an earlier age or its longer duration might result in cognitive benefits. If low vitamin A consumption occurs, the administration of supplementation ameliorates cognitive function. The level of *β*-carotene in the plasma lowers the risk of cognitive decline with age and the development of Alzheimer’s disease [90]. *β*-carotene intake exceeding 7.8 mg/day caused an improvement in cognitive function [90].

Recent findings indicate that *β*-carotene is locally metabolized into retinol and subsequently converted to retinoic acid in the hippocampus, a brain region where cognitive functions are impeded in early Alzheimer’s disease. Retinoic acid in the hippocampus plays a vital role in promoting neurogenesis and improving spatial memory deficits. A deficiency in vitamin A can result in a reduction in hippocampal volume and an increase in the deposition of amyloid-*β* (A*β*) plaques. Additionally, individuals with vitamin A deficiency tend to have lower levels of both retinol and its transport protein, retinol-binding protein 4, in their cerebrospinal fluid [90]. However, Flieger et al. consider it controversial to use carotenoids as Alzheimer’s disease biomarkers; the same applies to the prescription of *β*-carotene as a supplement in its prevention [90].

*β*-carotene might contribute to the delay of neurodegenerative ailment progression, suppressing pro-inflammatory cytokines, triggering Aβ peptide formation, and reducing oxidative stress. *β*-carotene is considered a promising Alzheimer’s disease antagonist since it possesses high binding energy with Alzheimer’s disease-related receptors (histone deacetylase and P53 kinase receptors) [5]. It should be noted that a statistically significant correlation between plasma *β*-carotene and Alzheimer’s disease, dementia risk, or cognitive decline was not confirmed in cohort studies. The beneficial impact of *β*-carotene in improving cognitive functions was demonstrated only among carriers of apolipoprotein E 4 alleles [90].

In an experimental study, researchers used a mouse model of Alzheimer’s disease. Mice were orally administered *β*-carotene at doses of 1.02 mg/kg and 2.05 mg/kg. After two weeks of treatment, the study revealed a reduction in acetylcholinesterase activity and a decrease in Aβ. Treatment of Alzheimer’s disease mice with 9-*cis*-*β*-carotene from the algae *Dunaliella bardawil* in the form of powder led to an enhancement in long- and short-term memory, as well as to a decrease in Aβ plaques, tau hyperphosphorylation, and neuroinflammation [90].

An age-related eye disease study which involved 3640 elderly AMD patients revealed a significant reduction in the risk of its late progression, of up to 25%, caused by daily supplementation with antioxidants (vitamin C 500 mg, vitamin E 400 IU, *β*-carotene 15 mg, and zinc oxide 80 mg) within a five-year follow-up period. The replacement of *β*-carotene with lutein and zeaxanthin supplementation, aimed at avoiding lung cancer risk, in an epidemiological 5-year trial did not provide a further decrease in risk in AMD patients [92].

A 10-year prospective trial engaging 37,846 males and females demonstrated that high dietary consumption of *β*-carotene (10 mg/day) reduced the risk of type 2 diabetes mellitus [5]. *β*-carotene supplementation within the carotenoid mixtures revealed a beneficial impact on adiposity in overweight and obese humans of all age groups, and a reverse correlation between circulating carotenoid plasma concentrations and body mass index or waist circumference, as indicators of obesity and obesity-associated metabolic ailments, was established [94]. *β*-carotene can upregulate adiponectin, which is suppressed in obesity and is known for its insulin-sensitizing, anti-inflammatory, and anti-atherogenic effects.

Carotenoids demonstrate promising benefits in overweight or obese individuals. A subgroup analysis of 18 observational investigations revealed a positive impact of *β*-carotene on weight loss [95]. *β*-carotene also exhibits hepatoprotective properties; its consumption in nutrition might decrease the risk of hepatic steatosis and injury generated by free radicals [81].

A negative correlation was demonstrated between the dietary intake of plant products rich in carotenoids and the incidence of several types of human cancer, including breast and endometrial cancers [54,97]. Higher serum concentrations of *β*-carotene were associated with a reduced risk of cervical cancer in Chinese women. Likewise, a notable connection was found between elevated serum *β*-carotene levels and a decreased risk of prostate cancer [54]. The beneficial effects of carotenoids and retinol in preventing estrogen receptor-positive cancers, such as breast and endometrial cancers, were demonstrated in vitro. This effect was achieved by inhibiting either 17 *β*-estradiol or aromatase. The anti-estrogenic action of *β*-carotene in breast and endometrial cancer cells is characterized by the inhibition of cancer cell proliferation caused by estradiol, as well as a reduction in DNA damage induced by catechol estrogens [97]. A meta-analytic investigation of the dietary consumption of *β*-carotene revealed a significant improvement in overall breast cancer survival, with a hazard ratio of 0.70 for the highest versus lowest intake and a hazard ratio of 0.93 per 1.2 mg/day increase due to supplementation when evaluating nutrition before diagnosis [99]. Although *β*-carotene has been proven to exhibit anti-carcinogenic action in various tissues (e.g., MCF-7 cells, U937, HL-60, myeloid leukemia, human adenocarcinoma colon cancer cells, human gastric cancer cell line) through different pathways, its high doses failed to possess chemopreventive effects in long-term clinical trials [86].

The protective network of the skin comprises numerous lipophilic and hydrophilic low-molecular-weight antioxidants, including carotenoids (*β*-carotene as well), vitamins, and some other substances [111]. Over time, these active principles are depleted, losing their protective impact on the skin. Topical application is an alternative measure to protect the skin from photo injury [88,100,112]. *β*-carotene levels remain constant from 30 to 60 min after UV-irradiation [34,100,113].

*β*-carotene is an important component of commercially available sunscreen protectants, both oral and topical (nutraceuticals and cosmeceuticals). Its efficacy as a systemic photoprotective agent depends on the dosage and duration of the administration. Synergistic effects between various antioxidants should also be considered to enhance the protection against ROS [94]. The administration of moderate doses of *β*-carotene (30 mg per day orally) before and during sunlight exposure might protect the skin against sunburns [94].

Basal skin protection might be enhanced systemically due to the use of *β*-carotene. The carotenoid level in skin and photoprotection efficacy observed in 12 women, reducing the intensity of erythema after 24 h of UV-mediated skin injury, was raised after 12 weeks of daily ingestion of 24 mg *β*-carotene and multi-carotenoid supplementation (*β*-carotene, lutein, and lycopene, each 8 mg per day). Such oral usage of *β*-carotene had an effect at the level of SPF 4 [92,114].

An eight-week oral intake of balanced natural plant antioxidants, including *β*-carotene, amongst others, possessed a long-term effect of elevating the carotenoid concentration in the stratum corneum [111]. After the completion of the supplementation course, the carotenoid level in the stratum corneum was lowered to its initial values within 5 weeks, which exceeds the 2–4-week period required for stratum corneum regeneration [96]. The topical application of *β*-carotene concerns the property of *β*-carotene to overcome the stratum cornea’s obstacle and impact deeper skin layers. The availability of *β*-carotene might be enhanced using medication-loaded nanostructured lipid carriers [115].

The topical application of a composition containing 0.2% *β*-carotene significantly increased human skin carotenoid levels by 1.75 times and demonstrated beneficial protective and antioxidant effects on the skin when exposed to IR radiation. Externally applied carotenoids as a single ingredient are less stable in the skin in comparison with those incorporated from dietary intake and accumulated in a mixture with various antioxidants [113]. A combination of antioxidants, including vitamin E, vitamin C, herbal extracts, and carotenes, applied externally demonstrated positive outcomes in extrinsic skin aging due to its widened neutralizing ability concerning oxidative stress [88,116].

An 8-week experimental model of ulcerative colitis-related local and systemic injury in hairless mice exposed to UV-irradiation revealed the ability of *β*-carotene to decrease an elevated degree of peroxidized cholesterol in the skin and matrix metallopeptidase 9 suppression and activity, along with the occurrence of wrinkles and sagging skin [117]. The administration of 60 mg *β*-carotene per day during 24–36 months to HIV patients in whom low plasma levels of carotenoids had been observed resulted in a significant growth in the number of CD4+ and CD8+ lymphocytes [54,85]. *β*-carotene administration in pregnant women did not significantly impact birthweight indicators, preterm birth, stillbirth, miscarriage, and fetal loss. Amongst HIV-positive females, supplementation acted protectively against low birth weight. Thus, maternal daily intake of *β*-carotene might possess a beneficial influence on maternal or infant mortality, although it may also cause some adverse reactions, increasing HIV transmission in some individuals as well [54].

The daily consumption of 50 mg of *β*-carotene for 10 to 12 years enhanced the activity of natural killer cells and reduced cancer development [85]. Other pathways involved in the immunological properties of *β*-carotene and its strengthening of the immune system comprise the regulation of the biosynthesis of prostaglandin E2, an immune suppressor [85], stimulation of thymus growth, increases in the number of T cells, IL-1, TNF-α, and mucosal immunoglobulin A, and an increment in cells with markers for IL-2 and transferrin activation [54].

It is believed that an intake of foods rich in *β*-carotene is highly suggested, seeing that it is related to a lower risk of chronic disorders and might provide the consumption of a sufficient quantity of antioxidants. A healthy diet, which consists of 100–500 g of fruit and vegetables per day, ensures a required amount of carotenoid-rich food [118]. The food additive E160a, of yellow to orange color, which is produced from carrots, is a mixture of carotenes or *β*-carotene. It is approved in the EU and the USA for food applications as an ingredient in soft drinks, juice, butter, some fruits and vegetables in vinegar or brine, jam, cheeses, candies, breakfast cereals, fats, sausages, pates, and bakery products. The food additive E160a is commercially produced from carrots [85].

The dietary reference intake for vitamin A is 0.9 mg, and it can be supplied by 10.8 mg of *β*-carotene per day. Generally, the acceptable daily intake of *β*-carotene is 7–15 mg [38]. Despite their various benefits, global dietary studies reveal an inadequate consumption of carotenoid-rich foods. In the US and UK, most subjects consume only 1–2 mg of *β*-carotene daily [114]. Although some regulatory agencies do not approve the recommended intake values, a plasma *β*-carotene level of 0.4 µmol per liter seems to support its preventive health potential and might be reached with a daily dose of 2–4 mg [114,118]. Notwithstanding the fact that the US National Cancer Institute and the US Department of Agriculture propose an intake of 3–6 mg of *β*-carotene for a reduced risk of chronic ailments [114], the NHANES survey demonstrated that the average daily consumption of *β*-carotene by Americans is 1.9 mg [38]. In most EU countries, the recommended consumption was approved in agreement with the assumption that 4.8 mg *β*-carotene is required to supply 800 micrograms of vitamin A [118].

In clinical studies, *β*-carotene was consumed in the range of 15–50 mg per day with good safety in intake concentrations. It has also been beneficially applied to treat inherited photosensitivity ailments at a dosage of 180 mg per day or even higher, without any adverse reactions, excluding hypercarotenemia. *β*-carotene is not a mutagenic, carcinogenic, embryotoxic, or teratogenic agent and does not give rise to hypervitaminosis A [54,118].

Considering some safety items associated with the prolonged administration of *β*-carotene alone, there occurs a tendency towards the application of its low doses in multi-carotenoid formulations compounded with other antioxidants for safe and more effective uses [114].

#### 3.2.3. Lycopene

Lycopene (C_40_H_56_, molecular weight 536.88 g/moL) is a red pigment found mainly in fruits and vegetables [119]. The chemical structure of lycopene contains a linear chain with 11 conjugated double bonds (Figure 1), which gives it its characteristic red color. It is a highly hydrophobic molecule.

The main plant sources of lycopene are tomatoes, pink grapefruit, and papaya. It is also produced by bacteria such as the *Blakeslea trispora* strain. Bacteria are mainly used for the industrial production of this dye [119]. In fresh tomatoes, lycopene is mainly found in the so-called ‘*all-trans*’ form, which is stable, while the ‘*cis*’ isomers are more stable. The ‘*cis*’ state of lycopene has a lower melting point, greater solubility in oil, and crystallizes less [119,120]. Interestingly, processed tomato products contain more lycopene that our bodies can use than fresh tomatoes [120].

People in various countries consume different amounts of lycopene, usually from cooked tomatoes, as it is better absorbed. In Finland, for example, the daily intake is around 0.7 mg, while in the United States, it ranges from 3.7 mg to 16.1 mg. It is worth mentioning that nanocapsules help in the targeted delivery of lycopene [121,122]. It is commonly used as a natural food coloring to enhance the visual appeal of food and provide a red color. Lycopene intake of up to 3 g/kg daily has no adverse effects [123].

Lycopene may have anti-inflammatory properties and be useful in treating chronic inflammation. It reduces oxidative stress and inflammation, which are key factors in the development of age-related diseases [119,124].

Supplementation with lycopene may improve cognitive function and reduce the risk of neurodegenerative disorders such as Alzheimer’s disease. The regular consumption of tomato products providing approximately 30 mg of lycopene per day over a period of several weeks has been demonstrated to engender improvements in oxidative stress markers, with the potential to support neuroprotection in older adults [125]. Lycopene is also recognized for its potential role in reducing the risk of certain cancers, with evidence supporting its efficacy against prostate cancer (where doses of 15–30 mg daily in humans, or 5–45 mg/kg diet in preclinical studies, administered over 8–12 weeks have shown significant protective effects) [126], lung cancer (where doses of 15–60 mg/day, particularly higher doses associated with a 3.4-fold increase in lung tissue concentration, have demonstrated benefits over durations of 8–14 weeks) [127], and stomach cancer (where daily intakes ranging from 5 to 10 mg for general protective effects to 15–30 mg for stronger preventive outcomes, sustained over several months, have been linked to significant reductions in risk) [128].

Lycopene has a positive impact on the cardiovascular system by lowering the risk of cardiovascular disease and improving lipid profiles. Daily doses ranging from 6 to 15 mg taken over 8 weeks have been shown to reduce oxidative stress, decrease inflammation markers such as high-sensitivity C-reactive protein, and enhance endothelial function. Higher doses, exceeding 25 mg daily, may lower LDL cholesterol levels by approximately 10%, which is comparable to the effects of low-dose statin therapy, as indicated by meta-analyses. Supplementation with cooked tomatoes, providing around 200 g daily over 60 days, has significantly enhanced antioxidant enzyme activity and reduced lipid peroxidation markers. Animal studies support these findings, demonstrating reductions in total cholesterol, LDL cholesterol, and atherosclerotic plaque area with doses equivalent to 10–15 mg/kg/day over 8–12 weeks [129,130]. It has been studied for its potential role in improving male fertility and supporting general sexual health [120].

Lycopene may protect against AMD in elderly people by exerting antioxidative and anti-inflammatory effects. Studies have demonstrated that pretreatment with 2 μM lycopene for 12 h significantly inhibited TNF-α-induced oxidative stress and inflammation in retinal pigment epithelial cells by suppressing NF-κB nuclear translocation and ICAM-1 expression. This protective mechanism involves the activation of the Nrf2 pathway, resulting in increased glutathione synthesis and a reduction in reactive oxygen species levels [131]. Lycopene possesses significant photoprotective effects and may be effectively used in skincare products, with in vitro studies demonstrating optimal effects at concentrations of 100 μM over 24 h to improve fibroblast proliferation and reduce reactive oxygen species (ROS) levels. In vivo studies using aged rat models showed that daily supplementation with 100 mg/kg lycopene for 8 weeks improved microvascular density, increased the collagen I/III ratio, and mitigated signs of photoaging by enhancing mitochondrial function and reducing markers of oxidative stress [132]. It has been shown to potentially reverse insulin resistance and promote microvascular neovascularization to protect aging skin [119,120]. Lycopene has also been suggested to reduce the risk of osteoporosis [133].

Lycopene has shown promise as an anti-inflammatory agent in animal studies. Research in the Wistar rat model indicates that lycopene may reduce the risk of developing type 2 diabetes [134]. Specifically, lycopene has been found to lower urine and blood glucose levels, increase serum insulin levels, and decrease the risk of diabetes-related pancreatic damage [135]. Specifically, it also organically reduces the risk of kidney damage [135]. In mice, a role in weight maintenance has been suggested [136]. In mice, there is also evidence suggesting that lycopene may play a role in weight maintenance.

In rabbits, lycopene supplementation at a dose of 5 mg/kg body weight/day for 4 weeks significantly reduced the concentration of LDL cholesterol by nearly 50% and improved the LDL/high-density lipoprotein (HDL) ratio from 4.36 ± 1.1 to 2.7 ± 1.2, compared to the placebo group, while HDL cholesterol levels remained unchanged. The intervention was administered in the form of lycopene beadlets mixed into a high-cholesterol diet [137]. Lycopene supplementation reduces oxidative stress and apoptosis in aging mouse oocytes, as demonstrated by the addition of 200 nM lycopene to in vitro maturation media over 48 h, which significantly lowered levels of oxidative markers such as hydrogen peroxide and malondialdehyde while enhancing antioxidant defenses, including total antioxidant capacity, reduced glutathione, and superoxide dismutase activity, thereby improving oocyte morphology and reducing fragmentation and degeneration [138]. It is used at doses ranging from 1 mg to up to 500 mg/kg body weight [119].

Laboratory studies suggest that lycopene may prevent the progression of aging by reducing inflammation through the prevention of IL-6 and IL-1β mRNA expression and the NF-κB pathway [139]. Several studies in cell lines have confirmed the protective effect of lycopene, with concentrations ranging from 0.5 to 25 µM, showing antioxidant properties by significantly reducing ROS levels and oxidative damage in as little as 3 to 24 h of exposure. Lycopene at 2 µM was particularly effective in lowering ROS production by approximately 40% in macrophages exposed to cigarette smoke extract, while higher concentrations like 10–25 µM demonstrated cytotoxic effects in certain settings [140,141,142].

Lycopene supplementation at doses of 3 and 6 mg/kg per day reduced endoplasmic reticulum stress in steatohepatitis by inhibiting the apoptosis signal-regulating kinase 1 and p-c-Jun N-terminal kinase signaling pathway in both in vitro and in vivo models [143]. The daily administration of lycopene (10 mg/kg) to high-fat-diet rats for 12 weeks led to a marked improvement in metabolic disturbances and a diminishing of obesity-induced NF-κB expression and inflammatory cytokines [144]. Recent studies suggest that lycopene may positively impact male fertility by enhancing sperm quality, regulating sex hormones and signaling pathways, and providing antioxidant protection [145]. In a study involving rats, the administration of lycopene (5 mg/kg intragastrically for 4 weeks) helped reduce testicular damage caused by lipopolysaccharide [146]. This improvement was linked to an enhanced lipid metabolism and a more effective inflammatory response.

Taking 10 mg of lycopene a day for 2 months may help with diabetes by boosting the body’s defenses against damage [147]. Another study involving 26 young volunteers showed that taking 5.7 mg of lycopene for 26 days significantly reduced the production of the inflammatory marker TNF-a [148]. 

Generally, more research is needed to fully understand the mechanisms and potential benefits of lycopene in age-related diseases, but studies to date are promising and provide clues as to its potential benefits.

### 3.3. Xanthophylls

#### 3.3.1. Fucoxanthin

Fucoxanthin is a highly abundant carotenoid pigment (C_40_H_58_O_6_, molecular weight 658.91 g/moL) constituting over 10% of the total carotenoid production in nature, particularly in marine environments [149]. It is an orange pigment present in the chromatophores of some macro- and microalgae as a major carotenoid. The primary source of fucoxanthin is the group of brown algae from the Phaeophyceae family [150]. Fucoxanthin was first isolated in 1914 in Germany from brown seaweeds [150]. Currently, fucoxanthin is widely used in sports supplements as it enhances fat-burning in white adipose tissue by increasing the activity of the protein thermogenin.

Algae-extracted fucoxanthin is a natural molecule (Figure 1) with diverse features and bioactivities, finding applications in the food, cosmetics, and pharmaceutical industries. Although it can now be produced by chemical synthesis, its oil extraction from algae is more accessible, safe, and utilized as fucoxanthin-containing beneficial substances without any additional purifications [151]. It absorbs green and blue light at 450–540 nm, giving algae an olive-brown color [149]. This compound has a unique molecular structure, featuring an allenic bond, a 5,6-monoepoxide, a highly conjugated system, and several functional groups such as hydroxy, epoxy, and carboxyl.

Fucoxanthin, due to its many conjugated double bonds, is vulnerable to light, oxygen, heat, and pH changes, making long-term storage difficult. Therefore, it is necessary to encapsulate fucoxanthin [152]. However, thanks to these features, fucoxanthin exhibits antioxidant properties [149]. In recent decades, numerous studies have documented a wide range of biological activities in fucoxanthin, including anti-inflammatory, cytoprotective, anticancer, anti-diabetic, anti-obesity, neuroprotective, and skin-protective [150]. Fucoxanthin improved hepatic steatosis caused by metabolic perturbations following palmitic acid-induced hepatic lipid deposition in experimental mice [153]. A diet rich in fucoxanthin may help reduce body fat accumulation and regulate glucose and insulin levels in the blood [150].

It should be mentioned that fucoxanthin is transformed into fucoxanthinol through hydrolysis by digestive enzymes such as cholesterol esterase and lipase in the gastrointestinal tract and absorbed by intestinal cells. Fucoxanthinol is considered its main active metabolite in humans [154]. Various scientific studies have shown that fucoxanthin and its metabolite fucoxanthinol can trigger G1 cell cycle arrest and apoptosis in different cell lines and inhibit cancer development in many animal models [154]. Fucoxanthinol is then further metabolized and converted to amarouciaxanthin A in the liver [149].

Numerous cellular and animal studies have shown that fucoxanthin and other carotenoids have anticancer effects. However, investigating their role through clinical trials is lacking [49]. In vitro studies demonstrated that fucoxanthin (doses 75–100 μM) effectively inhibited the proliferation and induced apoptosis in NCI-H1299 and A549 lung cancer cells [155]. Terasaki et al. [156] conducted in vivo investigations of the chemopreventive potency of fucoxanthin (30 mg/kg body weight during 14 weeks) and its influence on the gut microbiota in a model of colorectal adenocarcinoma. They found a substantial inhibition of the multiplicity of inflammation-associated colorectal cancer in mice.

Fucoxanthin may be a promising molecule for cancer therapy because it addresses many of the hallmarks of tumor cells [49]. Neumann et al. [157] revealed the antioxidative, anti-inflammatory, and antiproliferative effects of purified fucoxanthin derived from the diatom *Phaeodactylum tricornutum* in an in vitro study in blood mononuclear cells and various cell lines. It was found that fucoxanthin is selectively toxic to cancer cells and is a pro-oxidant molecule for them [158]. Treatment with fucoxanthin inhibits processes related to metastasis, including invasion, migration, the epithelial–mesenchymal transition, and angiogenesis. Additionally, fucoxanthin impacts DNA repair pathways, which may play a role in tumor cell resistance. Furthermore, combining fucoxanthin or its metabolite fucoxanthinol with standard anticancer treatments can enhance conventional therapies by reducing drug resistance [159]. Wang et al. [160] concluded that fucoxanthin can inhibit the growth of sarcoma in mice at doses of 50–100 mg/kg.

Utilizing nanoformulations enhances the storage and biocompatibility of fucoxanthin for a wide range of food applications [161,162]. Zhao et al. [162] investigated the use of short-chain glucans as a building block for developing a starch-based carrier to deliver fucoxanthin, a hydrophobic bioactive compound, to various segments of the intestine. The water solubility of fucoxanthin significantly improved when complexed with zein nanoparticles.

Epidemiological data and clinical trials using reconstructed human skin cultured with primary human keratinocytes and fibroblasts provide evidence that UV radiation is the primary cause of skin aging, inflammation, and cancer [163]. The influence of fucoxanthin after a 10-day culture of reconstructed human skin resulted in a stratified epidermis with well-defined stratum basale, spinosum, granulosum, and corneum.

The ethanolic extract of *Phaeodactylum tricornutum* demonstrated a high content of fucoxanthin (6.67 g/100 g of extract). The antioxidant activity and photostability of this extract against UV rays were evaluated using in vitro models of reconstructed human epidermis. The results showed that the extract reduced oxidative and inflammatory stress markers, as well as cytotoxicity and sunburn cells, while also demonstrating an excellent safety profile [164]. Fucoxanthin shows a protective effect against UVB-induced skin photoaging by significantly suppressing UVB-induced matrix metalloproteinase13, vascular endothelial growth factor expression, and epidermal hypertrophy in hairless mice [165]. Fucoxanthin exerted anti-inflammatory effects on keratinocytes and ameliorated atopic dermatitis symptoms by regulating innate lymphoid cells to normalize immune responses in a mouse model of atopic dermatitis [166].

Fucoxanthin has been shown to protect retinal pigment epithelium cells from premature aging caused by oxidative stress and to reduce the loss of photoreceptor cells in an in vivo model of AMD induced by sodium iodate [167]. Fucoxanthin pretreatment (10 mg/kg body weight for 2 weeks) reduced ROS generation and lipid peroxidation in a sodium iodate-induced retinal degeneration animal model. It was proven that fucoxanthin is non-irritant, and the use of alkyl benzoate as a solvent for fucoxanthin was suggested.

It was recently found that the antimicrobial activity of fucoxanthin is superior to that of astaxanthin [168]. Fucoxanthin was effective against a wide range of bacteria at low concentrations, ranging from 10 to 250 µg/mL. Toxicological studies on animals demonstrate that fucoxanthin is safe at a dose of 200 mg/kg body weight and higher.

The administration of fucoxanthin, which was extracted from the brown seaweed *Sargassum wightii*, significantly reduced blood pressure and the activity of the angiotensin-I-converting enzyme in diabetic rats with hypertension. Additionally, treating these experimental animals with fucoxanthin resulted in a notable decrease in their hyperglycemic state [169]. Furthermore, fucoxanthin also helped alleviate oxidative stress by preserving endogenous antioxidant levels in hyperglycemia rats. It is unique among other carotenoids in exerting anti-neurodegenerative effects against oxidative stress, amyloid protein aggregation, and neurotransmission dysregulation, as it can penetrate the blood–brain barrier [170].

Recently, López-Ramos et al. conducted a double-blind clinical trial involving 28 patients diagnosed with metabolic syndrome [171]. Participants received 12 mg of fucoxanthin daily for 3 months. After the treatment period, significant positive changes were observed, including reductions in body mass index and waist circumference, as well as improvements in triglyceride levels and total insulin secretion. Additionally, Kang et al. tested a wrinkle care cream containing 0.03% fucoxanthin on adult women, with the cream applied twice daily. No safety concerns regarding skin health were reported. After 8 weeks, an evaluation of wrinkles around the eyes revealed a statistically significant reduction in wrinkle depth.

Recently, experimental research highlighted the positive effects of a standardized extract of BrainPhyt, which contains 2% fucoxanthin from the microalgae *Phaeodactylum tricornutum*, on cognitive function in a mouse model of accelerated aging [172]. These included improvements in spatial working memory, long-term memory, and short-term memory. These benefits were linked to the regulation of oxidative stress and inflammation pathways.

Fucoxanthin has been proven to be a safe carotenoid with no side effects up to a dose of 2 g/kg body weight orally in experimental animals and at 0.5% application in human skin [163]. It was reported to be safe and non-toxic in mice up to a daily oral dose of 1 g/kg body weight (for 30 days) [173]. Tavares et al. [163] conducted in vitro investigations of the potential toxicity of fucoxanthin in two different solvents (alkyl benzoate and ethanol) which are frequently used in dermatological products. The study, performed on reconstructed human skin, demonstrated the non-irritancy of fucoxanthin. Furthermore, it ameliorated the detrimental effects of ethanol on skin tissue viability and the inflammatory response [163].

Generally, despite limitations such as chemical instability and poor bioavailability, fucoxanthin has garnered significant interest as a bioactive natural compound in the pharmaceutical and nutraceutical industries [174]. The primary challenges include a lack of extensive clinical studies on fucoxanthin, especially its anticancer potential [175].

#### 3.3.2. Lutein

Lutein (Figure 1) is a yellow pigment of the xanthophyll subgroup. The full formula of lutein is C_40_H_56_O_2_, with a molecular weight of 568.88 g/moL [176]. It is soluble in fats, organic solvents, and alcohols [176]. Therefore, eating high-fat meals has a positive effect on the bioavailability of lutein. Lutein is a nutrient most commonly found in dark green vegetables such as spinach, kale, parsley, brussels sprouts, lettuce, chives, pumpkin, avocado, broccoli, carrots, and egg yolk. In nature, it is most commonly found in the form of esters with fatty acids, which increases its solubility and bioavailability [176,177,178]. Since the human body cannot synthesize lutein de novo, it must be obtained through diet. Lutein is classified as ‘Generally Regarded as Safe’ and has minimal side effects even with long-term consumption [177].

The average daily intake of lutein is estimated to be 1.7 mg per day in the USA and 2.2 mg per day in Europe [179,180]. Absorbed from the diet in the stomach and duodenum, it forms complexes with monoglycerides and free fatty acids that allow it to be retained in water in the form of a micellar solution [178]. It is then transported to the liver, from where it is carried via lipoproteins to various tissues. Heat treatment increases its bioavailability.

Several studies have highlighted that lutein supplementation, typically at doses of 10–20 mg/day, has been shown to enhance its bioavailability in plasma and ocular tissues. Clinical interventions demonstrated that a daily intake of 10 mg lutein over 5 years was effective in reducing the progression of AMD. Higher doses, such as 40 mg/day for 9 weeks followed by 20 mg/day for up to 26 weeks, have also been tested without adverse effects, showcasing lutein’s safety for long-term use. Additionally, lutein intake in combination with other macular carotenoids for 8 to 12 weeks consistently improved macular pigment optical density and reduced oxidative stress in retinal cells [176,177,178]. Lutein is associated with mitigating age-related diseases and the aging of the body. In some studies, higher doses, such as 30–40 mg/day, were tested over shorter durations (e.g., 9 weeks) without significant adverse effects, demonstrating lutein’s efficacy and safety for long-term use in promoting healthy aging [179].

Lutein may help prevent some cancers [181] and cardiovascular disease [182]. Studies show that dietary supplementation of lutein at doses of 6–20 mg/day for periods ranging from 8 weeks to 5 years is associated with reduced risks of breast cancer and cardiovascular events, including coronary heart disease and stroke. In cancer prevention, lutein concentrations of 1–5 µM have demonstrated growth-inhibitory effects in breast cancer cell lines within 24 h in vitro. In cardiovascular disease, lutein has been shown to improve endothelial function and reduce markers of systemic inflammation when supplemented over extended periods. In the eye, lutein blocks blue light and has a photoprotective effect [177].

Lutein exhibits potent antioxidant and anti-inflammatory properties, with studies demonstrating that supplementation at doses of 10–20 mg/day for periods ranging from 12 weeks to 6 months effectively reduces oxidative stress markers and inflammatory cytokines, including TNF-α and IL-6, in both ocular and systemic conditions. In vitro experiments showed significant reductions in ROS and lipid peroxidation when lutein concentrations ranged from 5 to 20 µM, while clinical trials confirmed improvements in oxidative biomarkers and macular pigment density at doses of 10 mg daily over several months [183].

Short-term studies also demonstrate that doses as low as 5–10 mg/day over 12 weeks can enhance visual performance and retinal function, while higher doses up to 40 mg/day have been found to be safe in clinical trials [177,179,183].

Lutein reduces the negative effects of UV radiation and skin aging, with studies demonstrating that oral administration of 10–20 mg/day for 12 weeks significantly improves skin elasticity by 56% and hydration by 62%. When combined with topical lutein applications, these effects were further enhanced. Additionally, lutein-loaded lipid carriers demonstrated sustained release and improved stability, enhancing their photoprotective efficacy on the skin over longer durations [184]. Silk lutein (5–15 µM) effectively protected the keratinocytes from UV-mediated cell damage [185].

Lutein has a beneficial effect on the cardiovascular system, reducing the risk of heart disease. Thus, using its daily supplementation of 10–20 mg over 6 months to 2 years has been shown to improve vascular tone, reduce systemic inflammation, and enhance endothelial function, as well as lower oxidative stress markers associated with cardiovascular risk [182]. Lutein can play a role in maintaining brain health, as demonstrated in preclinical studies where doses of 50–100 mg/kg/day for 10 days significantly reduced neuroinflammation, oxidative stress, and neuron degeneration in the brain by modulating pro-inflammatory cytokines (IL-1β, IL-18) and reducing chemokines (macrophage inflammatory protein-2, cytokine-induced neutrophil chemoattractant) and inflammasome markers (caspase-1, etc.) [186].

Lutein also influences the lipid profile by reducing LDL levels and increasing HDL levels [187]. After a 2-week run-in period on a low-calorie diet, forty-eight participants aged 45 to 65 years were randomly assigned to receive either 20 mg of lutein per day or a placebo, in conjunction with a low-calorie diet, for 10 weeks. The study concluded that lutein supplementation, combined with a low-calorie diet, could enhance body composition and lipid profiles in obese individuals.

Lutein and zeaxanthin are powerful antioxidants known for their ability to prevent or reduce the risk of AMD [188]. A study investigated the visual effects of supplementing with lutein (10 mg/day) and zeaxanthin (2 mg/day) over 12 months. The results showed that the serum levels of these carotenoids significantly increased by the first follow-up visit at three months and remained elevated throughout the one-year intervention. Additionally, participants experienced significant improvements in chromatic contrast and photo-stress recovery time [189].

Recent in vitro studies have shown that lutein has antioxidant, antiviral, and anti-inflammatory properties and can protect cells from damage caused by oxidative stress [190]. Lutein can reduce inflammation by inhibiting the production of pro-inflammatory cytokines, with studies showing that daily doses of 10–20 mg for 12 weeks significantly lower levels of TNF-α, IL-6, and IL-1*β* through the suppression of NF-κB and mitogen-activated protein kinase (MAPK) signaling pathways, as observed in both in vitro and in vivo models [190,191]. Lutein has been shown to increase the proliferation and migration of human dermal fibroblasts at concentrations of 1–16 µmol/L, with time-dependent effects observed over 24 to 72 h, suggesting a potential role in wound healing through enhanced cellular regeneration and migration processes [192,193]. Lutein may protect retinal pigment epithelial cells against oxidative stress-induced cellular aging [193].

In vitro experiments have shown that lutein at concentrations of 10–50 µM modulates the expression of genes involved in lipid metabolism, with measurable effects observed within 24 to 48 h, suggesting a potential role in the prevention of non-alcoholic fatty liver disease through the regulation of lipid homeostasis and reduction in oxidative stress [194].

Numerous in vitro studies revealed that lutein can inhibit the activation of NF-κB, a key regulator of inflammation, suggesting its anti-inflammatory properties [183,186,190]. Lutein has been found to increase osteoblast differentiation and inhibit osteoclast formation in vitro, with studies demonstrating that concentrations of 10–50 µM applied for 24 to 72 h effectively promote bone health by reducing oxidative stress and suppressing nuclear factor of activated T-cells 1 and inflammatory cytokine expression, suggesting a potential role in promoting bone health [195].

In vivo, studies demonstrated that lutein supplementation at doses of 10 mg/day for 3 months significantly increased blood lutein levels from an average of 185.5 ng/mL to 430.3 ng/mL and enhanced macular pigment optical density, with levels gradually returning to baseline after the cessation of supplementation [196]. A higher dietary lutein intake in older adults was associated with higher plasma lutein concentrations [197]. Another in vivo study showed that lutein supplementation increased macular pigment optical density, which is an indicator of lutein levels in the macula of the eye [178]. In an animal model of AMD, lutein supplementation was found to protect retinal pigment epithelial cells from oxidative stress-induced damage [198]. Lutein (100 mg/kg, administered daily through an intra-gastric tube for 16 weeks) was found to be an effective hypolipidemic agent due to reducing the lipoprotein and triglyceride levels in hypercholesterolemic rats [199].

Several clinical trials investigated the effects of lutein supplementation with doses ranging from 10 to 20 mg/day for durations of 1 to 2 years. Significant reductions in inflammatory cytokines (e.g., TNF-α, IL-6), improvements in antioxidant capacity, and modulation of key genes involved in lipid metabolism and cellular stress responses were found [197,200]. Clinical trials also focused on improving cognitive function, with one trial demonstrating that lutein supplementation at 12 mg/day for 4 months significantly enhanced verbal fluency and learning efficiency in older women, suggesting its potential role in supporting cognitive health [201,202].

As is known, lutein plays a vital role in protecting the macula from light-induced oxidative stress. It is also the primary carotenoid found in the infant brain, where it contributes to cognitive development [203]. Research indicates that lutein supplementation through both maternal and infant consumption improves lutein levels in infants. Lutein supplementation has been studied in clinical trials for its potential role in the prevention and treatment of AMD. Clinical trials have shown that lutein supplementation can increase macular pigment optical density, which is associated with a reduced risk of AMD [197,198,200]. Lutein supplementation was found to increase macular pigment density and improve visual performance in low-light conditions [201].

A study of patients with non-alcoholic fatty liver disease found that lutein supplementation improved liver function and reduced liver fat [204]. Lutein supplementation has been studied in clinical trials for its potential role in the prevention and treatment of cancer, although more research is needed to determine its effectiveness [200].

Overall, clinical trials have investigated the use of lutein in combination with other nutrients or drugs to enhance its therapeutic effects in a variety of health conditions [205]. Lutein appears to have beneficial effects on many age-related diseases.

#### 3.3.3. Astaxanthin

Astaxanthin (C_40_H_52_O_4_, or 3,3′-dihydroxy-,*β*,*β*′-carotene-4,4′-dione, Figure 1) is a red-orange marine carotenoid with a molecular weight of 596.84 g/moL [206]. It is found inside a broad range of microorganisms and marine organisms, including yeasts, microalgae, plankton, krill, fish, and other seafood [207]. Astaxanthin brings about the typical color of salmon and crustaceans [208,209,210].

The presence of the hydroxyl and keto moieties on each ionone ring is considered to give unique properties to astaxanthin. This feature permits astaxanthin to be inserted into the membrane and stretched away into its entire width. In addition, astaxanthin has two predominant forms: the unesterified form is found in yeast or of synthetic origin and the esterified form occurs in algae [206]. In addition, the chemical structure of synthetic astaxanthin differs from the structure of natural astaxanthin. There exist different stereoisomers of synthetic astaxanthin, which can be dangerous for people [209].

When astaxanthin is ingested, it is absorbed in the duodenum. Before it enters the bloodstream, astaxanthin travels to the liver, where it binds to lipoproteins for distribution throughout the body. Due to its high lipophilicity, astaxanthin can also cross the blood–brain barrier, allowing it to reach the brain and eye structures. Additionally, it cannot be converted into vitamin A, which means that excessive intake is not result in hypervitaminosis A toxicity [208].

As is known, oxidative stress plays a significant role in the development of various diseases, including diabetes mellitus and cardiovascular diseases [211,212,213,214]. The key mechanisms through which oxidative stress contributes to these conditions include inflammation, worsening mitochondrial dysfunction, accelerated proliferation of vascular smooth muscle cells, apoptosis, and the accumulation of protein and lipid oxidation products.

Astaxanthin is believed to contribute to antiaging effects due to its antioxidant and anti-inflammatory properties [215]. These characteristics help prevent age-related muscle deterioration and enhance energy production in the mitochondria [216]. Additionally, astaxanthin can assist in eliminating free radicals generated during exercise and aerobic metabolism in the muscles [217]. Many studies demonstrate the antioxidant activity of astaxanthin, which quenches singlet oxygen, scavenges superoxide, hydrogen peroxide, and hydroxyl radicals, diminishes lipid peroxidation, and elevates such antioxidant enzymes as superoxide dismutase, catalase, and peroxidase [206,218,219,220].

Treatment with astaxanthin significantly diminished lipid peroxidation, both in basal conditions and in cell lines exposed to the pro-oxidant molecule tert-butylhydroperoxide in a neuroblastoma cell model. Moreover, in cells treated with astaxanthin, a significant increase in the expression of Nrf2, glutathione-disulfide reductase, and thioredoxin reductase was detected compared with control cells. Nrf2 is a transcription factor playing an important role in the activation of many cell protection genes involved in antioxidant defense [206,219]. In addition, the total amount of glutathione, the most abundant endogenous antioxidant molecule [221], significantly increased in cells incubated with 3.7 ng/µL of astaxanthin for 3 h compared with control cells. Therefore, astaxanthin has complex antioxidant activity, as it increases not only the level of glutathione but enhances the nuclear localization of Nrf2 and consequently enhances the expression of phase II antioxidant enzymes [206]. Antioxidant activity was evaluated for the nanoemulsion of astaxanthin, which reduced the ROS concentration in foreskin cells [222].

One more study confirms the antioxidant activity of astaxanthin in the oxygen and glucose deprivation model using the human neuroblastoma cell line. It noticeably decreased the content of intracellular ROS and malondialdehyde and upregulated superoxide dismutase expression; namely, astaxanthin attenuated oxygen and glucose deprivation-induced oxidative stress [219]. Oxygen and glucose deprivation increased the apoptosis of the human neuroblastoma cell line. Astaxanthin significantly influences the epigenetic expression of certain genes. Thus, adding astaxanthin to cells before oxygen and glucose deprivation inhibited the upregulation of Bax and the downregulation of Bcl-2 and decreased the enhancement of cleaved caspase-3. This study showed that astaxanthin has anti-apoptotic activity caused by oxygen and glucose deprivation [219].

Astaxanthin significantly downregulated the mRNA levels of inflammatory mediators (TNF-α, IL-1β, and high mobility group box-1 protein) induced by a hyperosmotic saline solution in human corneal epithelial cells [223]. Dietary astaxanthin in combination with α-tocopherol showed a synergistic inhibitory effect on oxidative stress in rats. The diet with astaxanthin elevated the level of α-tocopherol in the serum and liver. Additionally, the highest concentration of astaxanthin was observed in the α-tocopherol group. Moreover, the combination of α-tocopherol and astaxanthin noticeably reduced the levels of lipid peroxide in the serum of the tested animals [211].

Antihypertensive activity was revealed in spontaneously hypertensive rats treated with astaxanthin [212]. Higher levels of blood pressure and oxidative stress in serum with distinctly increased malondialdehyde content, reduced glutathione content, and decreased activities of superoxide dismutase and glutathione peroxidase were detected in the hypertensive animals. However, these indexes were the opposite in the group of spontaneously hypertensive rats treated with astaxanthin. Therefore, astaxanthin may attenuate hypertension via reducing blood pressure, mitigating oxidative stress, improving mitochondrial functions, and inhibiting the overactivation of the renin–angiotensin–aldosterone system in serum.

Astaxanthin was tested on a model of ochratoxin A-induced cardiac injury using male mice. In that study, the levels of Bax, caspase 3, caspase 9, malondialdehyde, glutathione, catalase, and superoxide dismutase in myocardial tissue were used as markers of apoptosis, oxidative stress, and inflammation. The levels of Bax, caspase 3, and caspase 9 were notably increased. There was a similarity between the index of lipid peroxidation for cardiac injury (malondialdehyde) and antioxidant markers of cardiac injury (glutathione, catalase, and superoxide dismutase). The levels of Bax, caspase 3, and caspase 9 decreased and the levels of Bcl-2, glutathione, catalase, and superoxide dismutase were increased in the astaxanthin group and the group treated with the combination of astaxanthin plus ochratoxin. This preclinical study can be a prerequisite for clinical trials with the usage of astaxanthin and its combination with other medicinal preparations for diminishing cardiac injuries induced by aging [224]. In addition, the study is in line with the investigation on the oxygen and glucose deprivation of human neuroblastoma cells concerning the inter-relation of Bax and Bcl-2 levels with astaxanthin [219].

Studies concerning the influence of astaxanthin on the development of cancer are quite important [225,226]. It was established that the nanoemulsion of astaxanthin in peanut oil caused apoptosis in human malignant melanoma in the lungs of mice after the injection of B16F10 cells. This nanoemulsion increased downstream representative apoptotic expressions of caspase-3, caspase-9, and ataxia-telangiectasia mutated kinase and decreased the levels of Bcl-2, cyclins D1 and E, nuclear factor κ-light-chain-enhancer of activated B cells, extracellular signal-regulated kinase 1/2, and matrix metalloproteinases. NF-κB facilitates tumor initiation/proliferation/development, increases angiogenesis, inhibits apoptosis, and resists drugs, causing the epithelial–mesenchymal transition, which helps distant metastasis [222].

Astaxanthin significantly diminished the levels of TNF-α, IL-1β, and high-mobility group box 1 induced by a hypertonic solution of sodium chloride in human corneal epithelial cells and the corneas of a hyperosmolarity murine model. Among these three groups, two groups received a 1 μL drop of 5 μM of astaxanthin 30 min before applying. Western blot analysis showed that after topical astaxanthin application, the key protein levels also were significantly downregulated compared with the control (only dropping of hyperosmotic saline solution in both eyes) [223]. Immune and non-immune cells can release them during infection or tissue damage [227]. Studies on the corneas of the hyperosmolarity murine model support using drops with astaxanthin for the prevention of dry eye disease.

As found in clinical studies, astaxanthin treatment reduced total cholesterol by 0.30 mM and low-density lipoproteins by 0.33 mM in prediabetes patients; therefore, astaxanthin treatment improved aspects of the pro-atherogenic lipid profile characteristic. The participants received either astaxanthin 12 mg daily or placebo for 24 weeks [228]. Therefore, astaxanthin as a diet supplement or in combination with synthetic medicinal products can improve lipid profiles in patients with a prediabetes state and dyslipidemia, considering that cardiovascular diseases and diabetes are important public health issues [207,214,229].

A clinical trial showed that a daily dose of 12 mg of astaxanthin for 12 weeks may help protect against cognitive decline related to aging in healthy older adults [230]. Supplementation with astaxanthin (4 mg/day) for 4 weeks was shown to rejuvenate the skin by reducing lipid oxidation and corneocyte desquamation in middle-aged individuals [231]. The oral administration of astaxanthin (100 mg/kg body weight for 16 weeks) to experimental mice significantly reduced UV-induced wrinkle formation and enhanced collagen fibers in the skin [232].

A recent study evaluated the antiaging effects of dietary astaxanthin extracted from *Haematococcus pluvialis* on the skin of aged mice [233]. The results showed that supplementation with 0.1% astaxanthin significantly improved the skin’s water retention capacity and viscoelasticity and reduced wrinkle formation, associated with intrinsic aging. These findings suggested that dietary astaxanthin could be a valuable ingredient in nutricosmetics aimed at combating chronological skin aging. In an in vitro study, Zhen et al. [234] examined the levels of ROS and antioxidant enzymes following pretreatment with particulate matter 2.5. The obtained results demonstrated that the pretreatment of the studied cell lines with astaxanthin (25 mg/mL) reversed the ROS levels and decreased the translocation of Nrf2. Generally, pretreatment with astaxanthin effectively decreased the ROS levels and improved the expression of antioxidant enzymes. Furthermore, astaxanthin protected cells from particulate matter 2.5-induced DNA damage. It was concluded that astaxanthin safeguarded keratinocytes from experimentally induced senescence by partially inhibiting excessive ROS generation through the NRF2 signaling pathway [234].

Thus, astaxanthin, as a natural antioxidant, can be used as a diet supplement or in combination with medicinal substances for reducing oxidative stress and inflammation, as well as the different health disorders caused by them.

#### 3.3.4. *β*-Cryptoxanthin

*β*-cryptoxanthin is recognized to be the fourth most abundant carotenoid in the USA [70]. Only a few fruits and vegetables (squash, persimmons, papaya, peppers, mango, apricot, tangerines, and oranges) are rich in *β*-cryptoxanthin. *β*-cryptoxanthin (C_40_H_52_O) (Figure 1) has a molecular weight of 552.9 g/moL. It is worth mentioning that *β*-cryptoxanthin draws the attention of scientists because of a positive correlation between *β*-cryptoxanthin intake and the prevention of various diseases [70]. In addition, *β*-cryptoxanthin belongs to provitamin A carotenoids [235]. *β*-cryptoxanthin is supposed to be absorbed into the intestine by two mechanisms: active transport utilizing enzymes such as scavenger receptor class B type 1 and passive diffusion. *β*-cryptoxanthin is more hydrophilic than lycopene, *β*-carotene, and *α*-carotene; therefore, it has relatively higher absorbability [236].

Sufficient carotenoid intake (zeaxanthin, lutein, *β*-cryptoxanthin, *α*-carotene, *β*-carotene, total carotenes, total xanthophylls, and total carotenoids) is considered to protect against age-related diseases by altering thioredoxin-interacting protein DNA methylation status in men [237]. *β*-cryptoxanthin showed anti- or pro-oxidant activity, depending on oxygen pressure, anti-inflammatory activity, reduced triglyceride content, and obesity [13,17,238,239]. Some sperm parameters are sensitive to the dietary intake of antioxidant nutrients, including *β*-cryptoxanthin [240].

*β*-cryptoxanthin showed its antioxidant activity in numerous in vitro tests, such as DPPH tests, tests with hydrogen peroxide, hydroxyl, and superoxide, lipid peroxidation tests, etc. [13]. Lower levels of lipid peroxidation were observed when UV radiation was used as an inductor of peroxidation [13]. In addition, these studies demonstrated the sun-protective properties of *β*-cryptoxanthin. Therefore, it can be concluded that this carotenoid might be one of the components of cosmetic products for the skin protection caused by sunlight.

Very interesting results were obtained in an in vitro study with human cervical carcinoma cells. They are related to the pro-oxidant properties of *β*-cryptoxanthin. *β*-cryptoxanthin induced cytotoxicity against human cervical carcinoma cells. This cytotoxicity is linked to many factors, including enhanced ROS generation, upregulation of pro-apoptotic genes (caspase-3,-7,-9, Bax, and p-53), simultaneous downregulation of anti-apoptotic Bcl-2, enhanced activation of caspase-3, and finally, cleavage of nuclei DNA [12]. In a study on human normal oral mucosal keratinocytes, *β*-cryptoxanthin suppressed the production of inflammatory cytokines (IL-6 and IL-8) and matrix metalloproteinases induced by 5-fluorouracil [241].

*β*-cryptoxanthin was found to inhibit oxidative stress-induced senescence in human renal tubular epithelial cells in a concentration-dependent manner [242]. After pretreatment with various doses of *β*-cryptoxanthin (0.1, 1, and 10 µM) on these cells in vitro, it was revealed to improve mitochondrial function due to promoting NRF2 nuclear translocation.

In an in vivo study, mice fed epigallocatechin and *β*-cryptoxanthin showed significant improvements in total lipids [17]. It could be indicated that such a combination of substances might be used for the development of functional products aimed at reducing obesity. One more study conducted on *Caenorhabditis elegans* fed *β*-cryptoxanthin showed a noticeable reduction in triglyceride content and body fat decrease compared with control-fed nematodes. Also, the study demonstrated that *β*-cryptoxanthin at a concentration of 0.025 µg/mL significantly increased worm survival after acute oxidative stress with 2 mM of H_2_O_2_. *Caenorhabditis elegans* is regarded as a model for studying obesity and aging [238].

One more study demonstrated the mitigation of non-alcoholic fatty liver disease under the influence of *β*-cryptoxanthin. It weakened high-refined-carbohydrate-diet-induced non-alcoholic fatty liver disease in both six-week-old male wild-type mice and *β*-carotene-15,15-oxygenase or *β*-carotene 9,10-oxygenase− double-knockout mice through different mechanisms in the liver–MAT axis, depending on the presence or absence of the *β*-carotene oxygenases mentioned above. This carotenoid also decreased the hepatic lipogenesis proteins acetyl-CoA carboxylase and stearoyl-CoA desaturase-1. The study suggested that *β*-cryptoxanthin influenced lipid metabolism differently depending on the presence or absence of carotenoid cleavage enzymes. *β*-cryptoxanthin suppressed lipogenesis and increased fatty acid *β*-oxidation in wild-type mice. These features of this carotenoid can contribute to diminishing liver steatosis in both genotypes [243]. Therefore, this carotenoid can be used in functional food products to delay the onset of obesity.

In a model of non-alcoholic steatohepatitis in mice, *β*-cryptoxanthin decreased inflammation and fibrosis mainly because of suppressing the increase and activation of macrophages and other immune cells and reducing oxidative stress [239]. *β*-cryptoxanthin alleviates myocardial ischemia/reperfusion injury because of inhibiting NF-kB-mediated inflammatory signaling in the rats. The elevated serum inflammatory cytokine (IL-1b, TNF-a, IL-6), and creatine kinase MB isoenzyme and lactate dehydrogenase levels were reduced in response to *β*-cryptoxanthin treatment in a dose-dependent way. It was found that 5, 10, and 25 mg/kg of *β*-cryptoxanthin elevated concentrations of IL-6, IL-1b, TNF-a, creatine kinase MB isoenzyme, and lactate dehydrogenase, which were significantly attenuated in response to *β*-cryptoxanthin treatment [244].

A recent in vivo study showed that *β*-cryptoxanthin reduces hyperglycemic-induced podocyte injury in diabetic kidney disease by enhancing Nrf2/heme oxygenase-1 signaling pathways. Mice received *β*-cryptoxanthin at a dosage of 10 mg/kg daily for 6 weeks [245].

Many carotenoids could reduce systemic oxidative stress that indirectly influences the macula [32]. The predicted plasma carotenoid scores for *β*-cryptoxanthin, *α*-carotene, and *β*-carotene were associated with a 25% to 35% lower risk of advanced AMD when comparing the extreme quintiles [32]. Therefore, it is essential to increase dietary consumption of a wide variety of fruits and vegetables that are rich in carotenoids to help reduce the incidence of advanced AMD.

In a 4-year longitudinal study, serum total carotenoids and *β*-cryptoxanthin were found to have low concentrations in biological fluids, which was associated with low lean body mass in older community dwellers [246]. In a clinical study of 17 postmenopausal obese women, all with a body mass index greater than 25 kg/m^2^, *β*-cryptoxanthin was administered at a daily dose of 4.7 mg for 3 weeks. This treatment significantly increased the serum levels of *β*-cryptoxanthin, which rose fourfold from 0.28 mg/mL to 1.15 mg/mL, and also elevated serum levels of high-molecular-weight adiponectin. It was noted that body mass index is negatively correlated with adiponectin levels in both sexes. Additionally, serum triglyceride levels showed a tendency to decrease by the end of the study, dropping from an average of 116 mg/dL to 100 mg/dL. Therefore, *β*-cryptoxanthin may be considered a potential agent for alleviating metabolic syndrome [247]. A longitudinal cohort study among middle-aged and older Japanese subjects demonstrated that the risk of the development of metabolic syndrome and abnormal lipid metabolism were inversely associated with the baseline serum *β*-carotene concentration and with serum α- and *β*-carotene, as well as *β*-cryptoxanthin concentrations, respectively [14].

Thus, the development of functional food or medicinal products containing *β*-cryptoxanthin for lowering rates of aging and avoiding different disorders caused by oxidative stress or inflammation is very important.

#### 3.3.5. Zeaxanthin

Zeaxanthin (C_40_H_56_O_2_, molecular weight 568.88 g/moL) or *β*,*β*-carotene-3,3′-diol is a xanthophyll carotenoid [248]. This pigment is responsible for the bright colors in many plants. It has a conjugated double-bond system, making it an excellent antioxidant. Zeaxanthin is structurally similar to its close relative, lutein, but differs in the hydroxy group at the 6′ position (Figure 1). Zeaxanthin has nine consecutive conjugated double and single bonds. It is its structure that gives zeaxanthin its intense orange-red color by absorbing specific wavelengths of light. It is the presence of conjugated double bonds that provides zeaxanthin with a powerful antioxidant effect. Zeaxanthin exists in three stereoisomeric forms, but only (3R,3′R)-zeaxanthin and (3S,3′S)-zeaxanthin have biological activity [249].

This compound is often taken specifically as a dietary supplement, especially for eye health. Understanding the natural sources of zeaxanthin is crucial for dietary planning and supplementation [67,250]. While corn, kale, and egg yolks are well-established sources of zeaxanthin, many other plant and marine sources also contain the carotenoid. Zeaxanthin is found in various orange, yellow, and red fruits and vegetables, including saffron, mangoes, papaya, broccoli, and green peas. *Zea mays* and its products, such as corn oil and meal, are good sources of zeaxanthin [251]. Saffron a spice derived from *Crocus sativus* flowers also contains zeaxanthin and crocin, among other bioactive compounds [252]; *Tagetes erecta* flowers, commonly used in the production of dietary supplements, are also a source of the carotenoid [253]. Some microalgae strains can synthesize zeaxanthin [254] and can be used to produce dietary supplements. Zeaxanthin content varies depending on cultivation methods, soil quality, and environmental conditions or post-harvest practices [255,256].

Zeaxanthin has significant antioxidant and anti-inflammatory properties and may play a role in cognitive function and brain health [183], and also arthritis and cardiovascular disease [252]. Zeaxanthin is concentrated in the macula of the eye, where it acts as a blue light filter, potentially preventing AMD [257]. It has also been shown to have potent effects on the central nervous system, improving cognitive function and protecting against neurodegenerative diseases such as Alzheimer’s disease [258]. The authors of one in vitro study showed that zeaxanthin inhibits the production of pro-inflammatory cytokines and enzymes. Zeaxanthin (0.5–5 µM) protected human retinal pigment epithelial cells against oxidative stress and inflammation induced by blue light [259].

In another study, zeaxanthin (1–10 µM) protected human skin fibroblasts from UV-induced oxidative damage [260]. It reduced UVA-induced oxidative stress and DNA damage, suggesting potential for skin photoprotection [261]. By inhibiting inflammatory pathways and cytokine production, zeaxanthin reduces chronic inflammation, a key factor in the development and progression of cancer. In vitro studies have shown that zeaxanthin modulates cell cycle progression and induces apoptosis in cancer cells [262]. It induces cell cycle arrest at various checkpoints, preventing the uncontrolled proliferation of cancer cells and promoting their death [263]. Zeaxanthin (5–20 µM) inhibited prostate cancer cell growth and migration [264].

Zeaxanthin also has the ability to absorb harmful UV rays, protecting cells from photooxidative stress and DNA damage [265]. For example, cultured skin cells supplemented with zeaxanthin showed reduced levels of UV-induced DNA lesions and apoptosis.

In vitro studies suggest that zeaxanthin and lutein may act synergistically to enhance antioxidant capacity and protect against oxidative stress-induced damage. The combination of the compounds showed a stronger protection of retinal cells against oxidative damage [266]. Additionally, combining zeaxanthin and vitamin E has been shown to enhance antioxidant defense mechanisms and also provide better protection against AMD [267,268].

In vivo studies involved administering zeaxanthin at a dose of 0.5 mg/kg body weight to mice daily for 12 weeks. The results showed improved insulin sensitivity and glucose tolerance, showing improvement in glucose metabolism and the potential to help treat diabetes [261]. Another study in mice showed a reduction in tumor growth and cancer prevention potential through induction of apoptosis [262]. The study was conducted in mice by administering zeaxanthin at a dose of 5 mg/kg body weight daily for 4 weeks. Zeaxanthin inhibited tumor growth and progression in a mouse model of colon cancer. Zeaxanthin (5 mg/kg body weight daily for 3 weeks) reduced inflammation and joint damage in a rat model of arthritis. It decreased the cartilage degradation, suggesting potential benefits for inflammatory conditions [269].

In vivo studies of zeaxanthin have also shown its neuroprotective effects, which is important for the treatment of neurodegenerative diseases [270]. It was found that 0.25 mg/kg body weight each of zeaxanthin and lutein daily for 4 months improved spatial memory and learning performance, suggesting the potential for cognitive health benefits [271].

Animal models of ischemic stroke induced by transient or permanent middle cerebral artery occlusion have also been used to determine the neuroprotective potential of zeaxanthin against cerebral ischemia–reperfusion injury [272]. The anti-inflammatory, anti-apoptotic, and vasoprotective effects of zeaxanthin contribute to its neuroprotective effects on ischemic stroke, preserving neuronal viability and function in the ischemic brain. Animal models of traumatic brain injury induced by controlled cortical impact or fluid percussion injury have been employed to investigate zeaxanthin’s neuroprotective effects against acute and chronic brain injury [273]. In vivo studies have shown that zeaxanthin supplementation attenuates neuroinflammation, blood–brain barrier dysfunction, and cognitive deficits in animal models of traumatic brain injury. The ability of zeaxanthin to modulate inflammatory pathways, reduce oxidative stress, and promote neurodegeneration contributes to its neuroprotective effects in traumatic brain injury, facilitating recovery and functional rehabilitation after brain injury [274].

Zeaxanthin also reduced weight gain and decreased adipose tissue inflammation, which is important in the treatment of obesity [275]. Animal models of diabetes, including rodents with streptozotocin-induced diabetes or a genetic predisposition to diabetes [276], have been used to evaluate the effects of zeaxanthin on glucose metabolism and insulin sensitivity. In vivo studies have shown that zeaxanthin supplementation improves glycemic control, increases insulin sensitivity, and reduces oxidative stress in diabetic animals. The results showed that zeaxanthin has a positive effect on glucose metabolism and diabetes-related complications [277].

In vivo studies have shown that zeaxanthin supplementation reduces plasma lipid levels, attenuates atherosclerotic plaque formation, and improves vascular function in hyperlipidemic and atherosclerotic animals [208]. The anti-inflammatory and lipid-lowering properties of zeaxanthin contribute to its cardioprotective effects, reducing the risk of cardiovascular disease in animal models. Studies have shown that zeaxanthin supplementation attenuates hepatic steatosis, reduces liver inflammation, and improves liver function in animals with non-alcoholic fatty liver disease or diet-induced liver damage [278]. Zeaxanthin has also been shown to have hepatoprotective activity. Zeaxanthin have been shown to suppress colon [279] and prostate [280] tumors caused by chemical carcinogens.

There are also several molecular docking studies of zeaxanthin docking to G protein-coupled receptors associated with inflammation and pain [281]. Zeaxanthin has been predicted to bind to potential anti-inflammatory and analgesic targets in silico, but this requires further study. Molecular dynamics modeling of the interaction of zeaxanthin with membrane lipids [276] showed that zeaxanthin absorbs free radicals, reducing singlet oxygen molecules, which confirms its antioxidant role.

Clinical trials have investigated the role of zeaxanthin supplementation in preventing or slowing the progression of AMD, a leading cause of vision loss in older adults. Randomized controlled trials have shown that supplementation with zeaxanthin, either alone or in combination with other carotenoids such as lutein and meso-zeaxanthin, can improve visual function and macular pigment optical density in individuals with AMD [257]. Zeaxanthin and lutein supplementation (10 mg combined) improved macular pigment optical density in early AMD patients [257].

Long-term supplementation with zeaxanthin has been associated with a reduced risk of developing advanced AMD and a slower progression of the disease in at-risk individuals [282]. Clinical studies have evaluated the effects of zeaxanthin supplementation on various aspects of visual performance, including contrast sensitivity, glare recovery, and visual acuity and also photoprotection against UV-induced damage [283]. Randomized controlled trials [284] have shown that zeaxanthin supplementation can enhance cognitive performance, memory, and processing speed in older adults with mild cognitive impairment or age-related cognitive decline.

Zeaxanthin is also available as a dietary supplement in capsules, tablets, and soft gels. Its recommended dose is usually 2–10 mg per day. Most commonly, it is prescribed to treat various eye conditions. However, the above-mentioned studies confirm the promising use of zeaxanthin in various treatment areas.

### 3.4. Clinical Trials on Carotenoids

Examples of completed clinical trials regarding the health-promoting properties of various carotenoids are presented in Table 1.

As can be seen from Table 1, most completed clinical studies have focused on the effects of carotenoids on the retina of the human eye [285,286,287], cancer [289,290,291] and skin health [293,294,295,296]. A clinical trial [297] revealed the positive effect of astaxanthin (8 mg per capsule, taken once a day for 12 weeks) on various factors, including markers of oxidative stress, inflammation, lipid levels, visual and skin health markers, and other indicators of longevity, mood, and skin function in healthy female participants.

A total of 180 subjects with early AMD were randomly divided into four groups to receive different treatments for 48 weeks [298]. These treatments included 10 mg of lutein per day, 20 mg of lutein per day, 10 mg of lutein plus 10 mg of zeaxanthin per day, and a placebo. The optical densities of the macular pigment were measured and analyzed at 24 and 48 weeks. The study concluded that supplementation with lutein and zeaxanthin could improve early functional abnormalities in the central retina of patients with early AMD. The clinical study was to assess the long-term effects of a fixed combination of lutein (10 mg), zeaxanthin (1 mg), omega-3 long-chain polyunsaturated fatty acids, and antioxidants on macular pigment optical density in patients with non-exudative AMD. After 12 months of intervention, the study found a significant increase in macular pigment optical density [299].

A 16-week clinical trial was conducted with 65 healthy female participants [300]. The participants in the study were given either a 6 mg or a 12 mg dose of astaxanthin, or a placebo. Conducted in Japan from August to December, the study aimed to evaluate effects during a time when environmental factors like UV exposure and dryness can worsen skin conditions. The findings suggest that long-term preventative supplementation with astaxanthin may help inhibit age-related skin deterioration and support skin health in conditions affected by environmental damage, likely due to its anti-inflammatory properties [300].

The health effects of carotenoids during pregnancy and lactation were reported by Zielińska et al. [301]. Carotenoids, especially lutein and zeaxanthin, play a crucial role in the development of vision and the fetal nervous system. They are essential for the development of the retina and contribute to energy metabolism and brain electrical activity. Additionally, emerging scientific evidence highlights the importance of carotenoids in preventing disorders in preterm infants, who are particularly vulnerable to conditions like retinopathy of prematurity.

Thus, the clinical studies presented in this section have demonstrated the most promising areas of possible application of carotenoids.

### 3.5. Adverse Effects and Safety Concerns of Carotenoids

There are limited data regarding the bioavailability and potential adverse effects of synthetic carotenoids in humans, and few studies have addressed this issue. According to [209], synthetic astaxanthin differs from its natural counterpart. Synthetic astaxanthin comes in various stereoisomers that do not occur in nature, and these isomers have poor bioavailability and lower technological stability. Furthermore, EC Regulation No. 1925/2006 [302] prohibits the use of synthetic astaxanthin in food products. In the United States, it is not generally recognized as safe because synthesized astaxanthin can pose risks to both humans and animals. Despite this, it is commonly utilized in the feed industry, particularly in aquaculture, as a coloring agent to produce pink and red hues.

Devaraj et al. [303] investigated the effects of synthetic lycopene at three different dosages: 6.5 mg, 15 mg, and 30 mg per day. A total of 82 subjects participated in the study, with 5 dropping out, leaving 77 who successfully completed the study. One participant receiving the 15 mg lycopene per day reported allergic skin reactions; however, no other participants experienced complaints related to bloating, diarrhea, or other gastrointestinal issues. The study did not indicate any serious adverse reactions associated with purified lycopene supplementation. Additionally, the findings demonstrated high bioavailability and good tolerability of synthetic lycopene across all three dosages. Importantly, the study found that a daily dose of 30 mg of lycopene could lead to approximately a 9% reduction in DNA damage (measured by a marker of mutagenesis) compared to baseline levels [303].

Metibemu et al. [110] detailed the pharmacokinetic and toxic properties of carotenoids. Carotenoids are absorbed like lipids and are transported to the liver via the lymphatic system. Their absorption is influenced by dietary factors; specifically, a diet rich in cholesterol enhances carotenoid absorption, while a diet low in cholesterol reduces it [110]. The topic of adverse reactions caused by carotenoids has also been addressed by Böhm et al. [31]. For example, the Panel on Additives and Products or Substances Used in Animal Feed established an acceptable daily intake of 0.034 mg of astaxanthin per kilogram of body weight. This guideline was based on a benchmark dose (benchmark dose lower confidence limit for a 10% extra risk) calculated from observations of liver hypertrophy in female rats during a chronic toxicity and carcinogenicity study, applying an uncertainty factor of 100 to the BMDL10 of 3.4 mg/kg body weight [304].

Although carotenoids exhibit protective effects at low concentrations, high doses (20–30 mg/day) may lead to pro-oxidative effects and toxic metabolic interactions. In smokers, *β*-carotene can degrade into reactive aldehydes and epoxides, which exhibit strong mitochondriotoxic effects, leading to a reduction in glutathione levels, lipid peroxidation, and impairment of the adenine nucleotide translocator, thereby disrupting ATP production [305]. High doses of *β*-carotene (>30 mg/kg body weight) may disrupt retinoid receptor balance, increasing cancer risk [306]. It was found that vitamin A and retinoids have several toxic effects on the redox environment and mitochondrial function [307].

Studies have shown that *β*-carotene degradation products at concentrations of 0.5–20 μM can inhibit mitochondrial respiration, induce mutagenic DNA damage, and increase oxidative stress. The mechanisms behind harmful effects remain unclear. During oxidative attacks, carotenoid breakdown products, which include highly reactive aldehydes and epoxides, are formed in the process of antioxidative action. *β*-carotene supplementation at a dose of 20 mg/day increased the risk of lung cancer in smokers, suggesting the need for caution when using high-dose supplementation [308].

Epidemiological studies indicate that high serum *β*-carotene levels may be associated with an increased risk of cardiovascular mortality in patients with type 2 diabetes [309]. In interventional clinical trials, *β*-carotene administration at doses of 15–50 mg/day for 1.4–12 years was associated with a slight but significant increase in cardiovascular mortality risk. Similar results were observed in the ATBC study, where supplementation with 20 mg/day for three years led to an increased risk of stroke in patients taking antidiabetic medications. An analysis including *α*-carotene, *β*-cryptoxanthin, lycopene, lutein, and zeaxanthin did not show a significant association between their serum levels and cardiovascular mortality risk, suggesting a potential specificity of *β*-carotene as a risk factor in T2D patients [309]. *β*-carotene supplementation at doses of 20–30 mg/day in smokers was associated with an increased risk of lung cancer (2.0% vs. 0.9% in the control group) in the AREDS study and a 46% higher lung cancer mortality in the CARET study (30 mg *β*-carotene + 25,000 IU vitamin A per day) [310]. At high concentrations (>5 mM), *β*-carotene may also interfere with the regeneration of α-tocopherol and ascorbic acid, weakening their antioxidant effects and leading to increased oxidative stress [305]. While carotenoids in moderate amounts may exert protective effects, high doses, particularly of *β*-carotene, may lead to pro-oxidative effects, increased lung cancer risk in smokers, and elevated cardiovascular mortality in patients with type 2 diabetes, highlighting the need for caution in supplementation [309].

Synthetic crystalline lycopene is a widely used nutritional supplement. Studies found that lycopene supplementation did not cause direct maternal or developmental toxicity in rats or rabbits, even at dosages as high as 3000 mg/kg/day [311]. Astaxanthin-containing preparations also have become increasingly popular as health food supplements. Therefore, evaluating its maximum safe daily intake is crucial for setting appropriate dosage levels [312]. Currently, there are discrepancies in the recommendations provided by different regulatory authorities, with suggested doses varying from 2 mg to 24 mg in different countries. The European Food Safety Authority recently proposed an acceptable daily intake of 2 mg based on a toxicological study conducted on rats using synthetic astaxanthin. However, it is important to note that synthetic astaxanthin is not chemically identical to its natural counterpart [312]. Thus, further studies are recommended to investigate the safety differences between the two forms.

Thus, the study of the toxicity of carotenoids is primarily concerned with *β*-carotene. Some studies address the possible toxic effects of the synthetic carotenoid astaxanthin.

## 4. Conclusions and Future Perspectives

Carotenoids are tetra-terpenoid pigments produced by various plants, bacteria, and fungi. They are classified into two main groups: xanthophylls, which contain oxygen, and carotenes, which are purely hydrocarbon structures. Notable examples of carotenes include *α*-carotene, *β*-carotene, and lycopene, while lutein, astaxanthin, *β*-cryptoxanthin, fucoxanthin, and zeaxanthin are major representatives of the xanthophyll category.

Carotenoids possess various health-promoting properties and are considered promising candidates for treating age-related diseases and improving skin conditions. By reducing oxidative stress, carotenoids may help alleviate the inflammation and cellular dysfunction associated with numerous health conditions. They can inhibit the development of AMD, prevent cancer and neurodegeneration, reduce metabolic disorders, and enhance skin appearance, among other benefits. Therefore, developing functional foods or medicinal products that contain carotenoids is essential for slowing aging rates, preventing various disorders, and treating patients suffering from diseases linked to oxidative stress or inflammation. However, more extensive and conclusive clinical trials are necessary to confirm the efficacy of carotenoids in specific health conditions. Further research is also needed to determine optimal dosages and potential side effects.

To ensure the optimal delivery and bioavailability of carotenoids, encapsulation in vegetable oil-based liposomes is a potential solution due to the presence of long-chain aliphatic molecules. Because carotenoids have low bioavailability due to their lipophilicity and low stability resulting from their conjugated double bonds, nanoscale delivery systems have recently been proposed for controlled delivery to targeted areas. When considering safety, it is often recommended to use low doses of carotenoids when administered alone. Multi-carotenoid formulations and other antioxidants may promote safer and more effective usage. Carotenoids exhibit synergistic effects with other nutrients, particularly other carotenoids, and antioxidants, enhancing their bioactivities and overall health benefits. The primary challenges that remain include a lack of extensive clinical studies on some carotenoids and their interactions with other drugs. Today, it is appropriate to utilize more integrative approaches for assessing a wide range of health aspects related to carotenoids, including multi-omics techniques, particularly transcriptomics, lipidomics, proteomics, and metabolomics. These assessments are conducted using various biospecimens, such as plasma, blood cells, urine, stool, etc.

In summary, while more research is needed to fully understand the mechanisms and potential benefits of carotenoids in age-related diseases, current studies are promising and provide valuable insights into their potential advantages.

## Figures and Tables

**Table 1:** Examples of completed clinical trials regarding the antiaging properties of various carotenoids (from ClinicalTrials.gov).

Official Title	Clinical Trials.gov ID	Location	Condition	Protocol (Study Type)	Subjects (Ages Eligible for Study)	Duration	Intervention/Treatment	Reference
Carotenoid Supplementation and Normal Ocular Health	NCT02147171	Manchester, UK	Older human eyes	Interventional (phases 2 and 3)	88 (50–90 Years)	November 2011–April 2014	Dietary supplement: VisionAce containing carotenoids. Lutein-containing ‘VisionAce’ daily for a period of 1 year	[285]
The Effects of Lutein and Zeaxanthin Supplementation on Vision in Patients With Albinism (LUVIA)	NCT02200263	Baltimore, USA	Macular pigment and visual function in albinism	Interventional	10 (12 Years and older)	November 2014–April 2018	Lutein plus zeaxanthin. Participants received 20 mg of lutein plus 20 mg of zeaxanthin per day (for the duration of 12 months)	[286]
The Zeaxanthin and Visual Function Study (ZVF)	NCT00564902	North Chicago VA Medical Center	Atrophic age-related macular degeneration	Interventional	60 (45 Years to 90 Years)	October 2007–June2009	Dietary supplement: 8 mg of lutein and 8 mg of zeaxanthin administered during 12 months	[287]
Effect of BrainPhyt, a Microalgae Based Ingredient on Cognitive Function in Healthy Older Subjects (PHAEOSOL-THREE)	NCT04832412	Cork, Ireland	Cognitive function of healthy older subjects	Observational	66 (healthy males and females, 55–75 Years)	April 2021–May 2023	Dietary supplement: BrainPhyt (natural extract of *Phaeodactylum tricornutum* containing fucoxanhin) Doses: 2 capsules of 275 mg BrainPhyt for 24 weeks	[288]
Beta-carotene and Alpha-tocopherol Chemoprevention of Second Primary Malignancies in Head and Neck Cancer Patients	NCT00169845	Quebec City, Canada	Malignancies in head and neck cancer patients	Interventional (phase 3)	540 (18 Years and older)	October 2014–March 2018	Dietary supplement: alpha-tocopherol and beta-carotene (daily supplementation of one capsule of 400 IU dl-alpha-tocopherol) and one capsule of 30 mg beta-carotene (for 3 years after the end of radiation therapy)	[289]
Correlation Between Skin Carotenoid Levels and Previous History of Skin Cancer	NCT00836342	Boston, USA	Skin malignancies, including non- melanoma skin cancer	Observational	81 (50–75 Years)	February 2009–January 2012	Mean carotenoid levels in subjects with a history of squamous-cell carcinoma were compared to mean carotenoid levels in control subjects without a history of non-melanoma skin cancer	[290]
Lycopene in Treating Patients Undergoing Radical Prostatectomy for Prostate Cancer	NCT00450749	USA	Prostate cancer	Interventional (phase 3)	10 males (12 Years and older)	February 2008–May 2010	Total tissue lycopene concentrations in radical prostatectomy specimens in participants receiving 4–7 weeks of preoperative supplementation with lycopene (60 or 30 mg/day)	[291]
The Effect of Astaxanthin on Oxidative Stress Indices in Patients With Polycystic Ovary Syndrome	NCT03991286	Tehran, Iran	Polycystic ovary syndrome	Interventional	48 Females (18–40 Years)	January 2020–April 2021	Dietary supplement: Astaxanthin (8 mg), a 40-day course	[292]
Evaluation of the Benefits of FloraGLO™ Lutein on Skin Health	NCT03769779	Port Chester, New York, USA	Healthy skin elasticity and hydration	Interventional	60 (females, 30–65 Years)	June 2019–Marh 2020	Dietary supplement: FloraGLO lutein (20 mg) (after 42 and 84 days of use)	[293]
Effects of Isoflavone Combined with Astaxanthin on Skin Aging	NCT02373111	Seoul, Republic of Korea	Skin photoaging	Interventional	90 (45 Years and older females)	March 2015–December 2015	Dietary supplement: isoflavone Dietary supplement: astaxanthin. Each subject takes one tablet per day for 24 weeks. Each tablet contains isoflavone 27 mg and astaxanthin 4 mg	[294]
Astaxanthin (2 mg) + Lycopene (1.8 mg) + D-Alpha-Tocopherol (10 IU) For The Treatment Of Skin Aging	NCT03460860	Manila, Philippines	Healthy skin	Interventional	100 (Healthy individuals age 30 to 60 years old)	March 2018–July 2019	Dietary supplement: Astaxanthin (2mg)+Lycopene (1.8mg)+D-Alpha-Tocopherol (10IU): once daily, for 12 weeks	[295]
The Effect of 12-Week Dietary Intake of Lutein on Minimal Erythema Dose and Other Skin Parameters (VIST Lutein)	NCT03811977	Ljubljana, Slovenia	Skin erythema	Interventional	30 Females (25–55 Years)	March 2019– August 2019	Dietary supplement: lutein syrup: 12-week dietary supplementation (20 mg lutein/day)	[296]
A Study to Evaluate the Effect of Astaxanthin in Healthy Participants	NCT05376501	Richardson, Texas, USA	Healthy females	Interventional	60 females (18–50 Years)	June 2022–October 2022	Dietary supplement: Astaxanthin (8 mg per capsule) Time frame: 4 and 12 weeks	[297]
